# HyTC-Net: A Multi-Scale, Multi-Branch Hybrid Attention Cloud Detection Network for High-Resolution Image Cloud Texture Recognition

**DOI:** 10.3390/s26216694

**Published:** 2026-10-22

**Authors:** Yuqing Zhang, Hai Tan, Haoran Zhai

**Affiliations:** 1 Land Satellite Remote Sensing Application Center, Ministry of Natural Resources (MNR), Beijing 100048, China; zhangyuqing@lasac.cn (Y.Z.); 2@by2studio.cn (H.Z.); 2 School of Geomatics, Liaoning Technical University (LNTU), Fuxin 123000, China

**Keywords:** cloud detection, high-resolution images, convolutional neural networks, cloud texture recognition

## Abstract

**Highlights:**

**What are the main findings?**

- Introduces HybridTexture-aware Cloud Detection Network (HyTC-Net), a hybrid CNN–Transformer architecture that integrates a novel Cloud Texture Attention (CTA) module to specifically capture multi-scale cloud textures and frequency characteristics.
- Outperforms the evaluated comparison models on the ZY-3 satellite imagery dataset, demonstrating significant advantages, particularly in challenging scenarios such as thin clouds, fragmented clouds, and distinguishing clouds from snow.

**What are the implications of the main findings?**

- This method offers a more accurate solution for pre-processing high-resolution satellite data with limited spectral information.
- By effectively addressing challenges like blurred boundaries and texture variability, the method facilitates the execution of subsequent tasks.

**Abstract:**

In response to issues with high-resolution imagery from the ZY-3 satellite—such as blurred cloud boundaries and incomplete segmentation of thin clouds following training on the OmniCloudMask (OCM) baseline model, as well as the tendency for clouds detected against high-brightness backgrounds such as snow-covered terrain to blend with the background—this paper proposes a hybrid CNN–Transformer network, HyTC-Net, designed to enhance cloud texture recognition capabilities. Based on the ConvNext-Tiny backbone, this model innovatively incorporates the CloudTextureAttention (CTA) module and a lightweight Transformer module, LightweightMobileViT (LM), while combining ASPP with a deep supervision (DS) mechanism to enhance the perception of cloud textures and boundaries, thereby improving segmentation capabilities for complex scenarios such as thick clouds, thin clouds, clouds in high-brightness scenes, and patchy clouds. On the ZY-3 dataset, HyTC-Net achieved accuracy of 94.73 ± 0.18% and a precision of 92.28 ± 0.21%, significantly outperforming the baseline model OCM by 1.12 percentage points in accuracy and 2.4 percentage points in precision. Compared with mainstream models, such as UNet and DeepLabV3+, the accuracy improved by up to 6.6 percentage points and the precision by up to 17.9 percentage points. The results demonstrate that HyTC-Net can effectively address challenges arising from uneven textures in high-resolution imagery and limited spectral information, such as unclear boundaries, misclassification of highly reflective features, and failure to detect thin clouds.

## 1. Introduction

With the rapid development of space-based remote sensing technology in China, the demand for high-precision, high-resolution satellite image processing is gradually increasing. The ZY-3 satellite is China’s first civilian high-resolution stereoscopic mapping satellite; it was launched on 9 December 2012. It carries four sets of optical cameras, including one orthorectified panchromatic time-delayed integration charge-coupled device TDICCD camera with a ground resolution of 2.1 m; two forward-and backward-looking panchromatic TDICCD cameras with a ground resolution of 3.6 m each; and one orthorectified multispectral camera with a ground resolution of 5.8 m [1]. The ZY3-01 and ZY3-02 satellites, positioned 180° apart in the same orbit, form a true constellation, significantly enhancing data collection efficiency and spatial information acquisition capabilities. They provide crucial support for land resource surveys, as well as surveying and mapping services, and are of great significance to the development of China’s geographic information infrastructure [2]. In order to detect anomalies such as cloud cover for subsequent analysis, an automated approach is required to pre-process such a vast volume of data. Cloud detection is one of the key pre-processing steps in satellite image processing; the presence of clouds hinders the accurate analysis of remote sensing satellite images, as they obscure ground targets, preventing them from being observed by the satellite [3]. In recent years, numerous methods for cloud detection have been developed. Early approaches focused primarily on thresholding techniques, traditional statistical models and radiative transfer techniques; the main methods include the Normalized Difference Vegetation Index (NDVI), Random Forests and RTM-based inversion methods. Although each algorithm relies on the implicit or explicit inversion of the radiative transfer equation, the fourth category of inversion methods attempts to simultaneously invert all major components in complex scenarios. This differs significantly from satellite-based techniques, as it relies on ground-based measurements, which are typically carried out manually, rather than by computer. Initially, people tended to assume that surface observations, particularly those of total cloud cover, were accurate; however, due to differences in observational geometry, they may differ from satellite-based inversion results [4]. With regard to the thresholding method, a new approach known as the Fmask function has been proposed for the detection of clouds and cloud shadows in Landsat imagery. Fmask first uses rules based on cloud physical properties to distinguish between potential cloud pixels and clear-sky pixels; it then combines normalized temperature probability, spectral variation probability and brightness probability to generate cloud probability masks for land and water surfaces [5]. Subsequently, improvements were made to Fmask; the literature [6] details the development of the Fmask 4.0 algorithm, which primarily introduced several enhancements to the previous Fmask algorithm, incorporating the concept of historical time series, and was subsequently validated. In the early stages, China drew on international methods, but placed greater emphasis on domestic satellites. To address the issue of thin cloud cover, methods based on the Normalized Difference Vegetation Index (NDVI) and reflectance thresholds were employed; however, these approaches still lagged behind those of other countries. Subsequently, deep learning methods began to attract widespread attention, and a 2015 paper [7] proposed a deep learning model based on the U-Net framework. A 2019 paper [8] proposed RS-Net, a deep learning model based on the U-Net architecture, for cloud detection in optical satellite imagery, which improved the accuracy of cloud detection against a snow and ice background. To improve the detection of thin and patchy clouds, a cloud detection architecture combining Swin-Transformer [9] and U-Net was proposed in 2021, which not only enhanced the ability to detect thin and patchy clouds, but also reduced the likelihood of false positives and false negatives. In 2022, a paper [10] described the LSANET model, which utilized a self-attention mechanism to effectively enhance the processing of thin clouds and improve processing efficiency. The Swin-TransformerV2 model [11] can process images with a resolution of up to 1536 × 1536 and has set new performance records on visually representative tasks. Subsequently, a multi-branch convolutional attention network, MCANet [12], was proposed; this network is capable of efficiently extracting local and global information from images. In 2024, the core selective scanning mechanism of the Vision Mamba model [13] exhibited linear computational complexity when processing sequence data. Consequently, Vim’s computational complexity grows more slowly than that of the Swin-Transformer model, and it possesses a global receptive field, giving Vim a theoretical advantage in tasks requiring an understanding of overall structure and long-range dependencies. In 2025, a paper [14] proposed a deep learning network model for OCM, a variant of a convolutional neural network based on the U-Net architecture. Its lightweight design stood out, and its generalization ability was enhanced through Z-score normalization and mixed-resolution training. It is currently one of the more advanced neural networks and serves as the baseline model for the experiments in this paper. However, the OCM model does not perform as well as might be hoped when applied to the second-classification data from the ZY-3 satellite, with some cases of missed detections and false positives occurring. This is due to the poor cloud detection performance resulting from the lack of short-wave infrared and thermal infrared bands in the ZY-3 high-resolution satellite imagery [15]. Furthermore, although high-resolution optical satellite imagery offers extensive coverage and a wealth of ground feature data, it is susceptible to factors such as lighting and obstruction [16].

This paper proposes a method for cloud detection in high-resolution imagery from the ZY-3 satellite using cloud texture attention. It employs the ConvNext-Tiny backbone, which differs from OCM and enables higher model accuracy, and builds upon ConvNeXt, significantly improving segmentation accuracy through an optimized hierarchical representation [17]. The model also incorporates a lightweight Transformer enhancement LM module and a custom CTA module featuring a texture attention mechanism, which enhances the model’s ability to perceive cloud textures in high-resolution imagery. This enables it to segment clouds and snow with greater precision when analyzing high-resolution satellite imagery such as that from the ZY-3 satellite, which has limited spectral information. In the model, the lightweight hollow-space pyramid pooling ASPP serves as the bottleneck layer. The ASPP architecture consists of three sets of dilated convolutions, each with a different dilation rate, while the size of the convolution kernels is 3 × 3 in all cases. Unlike traditional fully connected layer pooling methods, the ASPP architecture utilizes hollow convolution and pooling operations, an approach which effectively reduces the number of parameters, thereby improving the model’s computational efficiency and generalization ability [18]. The HyTC-Net model is able to capture context more effectively, process features from the deepest layers of the image, and finally fuse these features in the decoder. Furthermore, the model’s DS enhances quality, which aids in refining cloud edges, ultimately yielding cloud detection results.

The main contributions of this study are as follows:

1. The CTA module with complementary feature branches. CTA integrates channel recalibration, multi-scale spatial texture attention, and local statistical modulation. The statistical branch provides a spatially aligned descriptor of local feature fluctuations, with a frequency-related interpretation based on aggregate non-DC energy.
2. A framework combining local texture representation with global context. HyTC-Net integrates CTA with LM modules and multi-scale contextual aggregation to address cloud–background ambiguity and cloud structures of different sizes in spectrally limited high-resolution imagery.
3. An evaluation of segmentation performance and component behavior on ZY-3 imagery. Comparative experiments, scene-specific evaluations, module and loss ablations, and attention visualizations examine the proposed framework. An additional inference-time intervention assesses the sensitivity of predictions to F-branch modulation.
4. The loss configuration for region and boundary optimization. TAB is described as a modified form of the original Boundary loss, whereas DT is described as an improved loss based on Dice and IoU terms. Their combination is designed to improve region overlap and boundary delineation.

## 2. Materials and Methods

### 2.1. HyTC-Net Model

**Figure 1:**
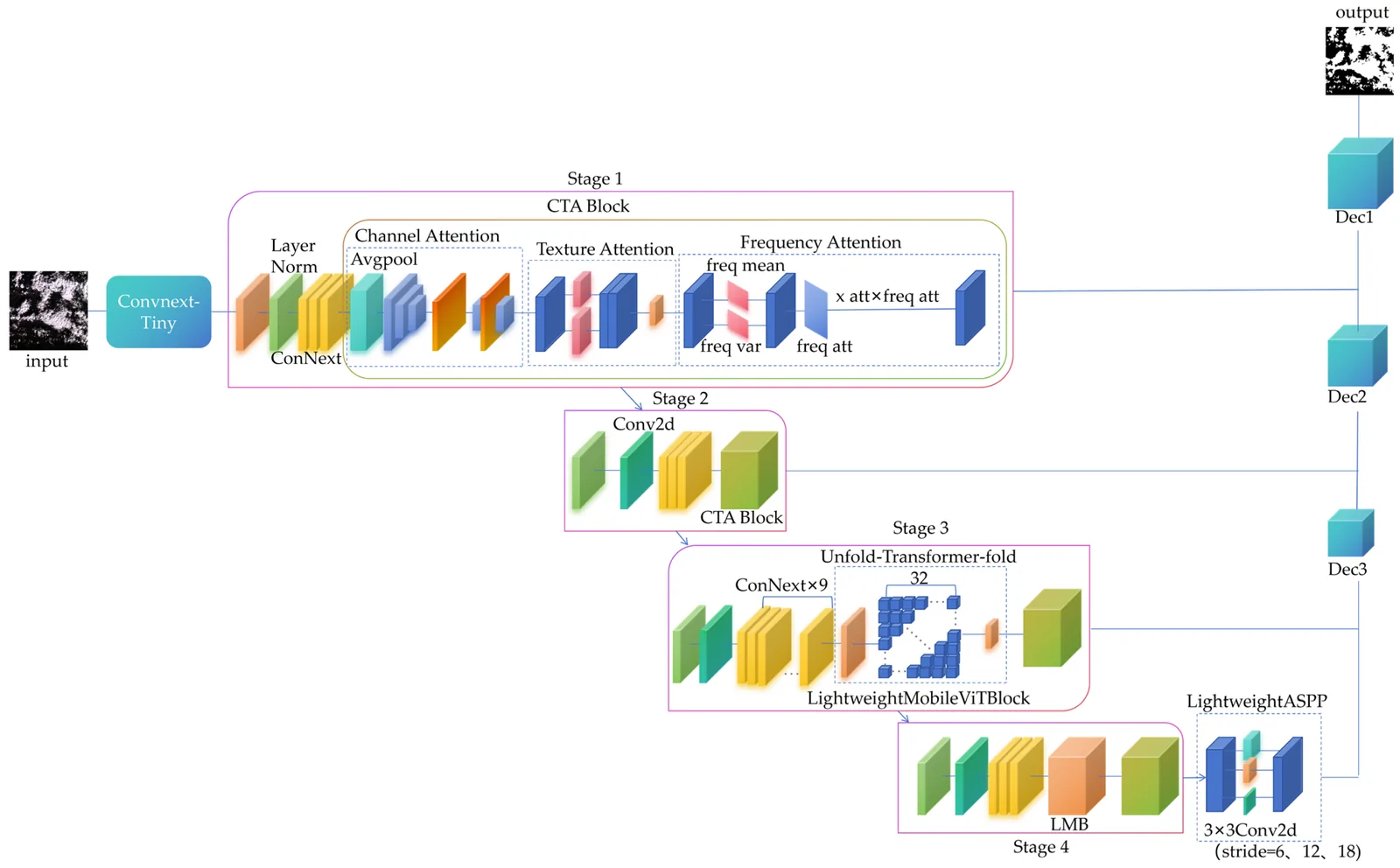
Overall architectural diagram of the HyTC-Net model.

The OCM model proposed at this stage employs a U-Net-based architecture when processing the binary classification data from the ZY-3 satellite, with Edge Next-Small serving as the encoder backbone to extract image features. At the same time, the model enhances its adaptability to different sensors through dynamic Z-score normalization. The Edge Next-Small backbone of the OCM model introduces a split-depth transposed attention encoder, which divides the input tensor into multiple channel groups and utilizes deep convolutions and cross-channel self-attention mechanisms to implicitly expand the receptive field, thereby encoding multi-scale features [19]. The backbone parameters of this model are smaller and more lightweight, combining the strengths of convolutional networks and Transformers for the feature extractor. The decoder employs a U-Net architecture that fuses edge, texture and semantic features to improve segmentation accuracy. The data undergoes a series of enhancements, including dynamic Z-score normalization, which plays a significant role during the data processing stage without substantially increasing memory and processing requirements. Dynamic Z-score normalization is a normalization strategy that helps avoid outlier issues and can adapt to different sensors [20]. The HyTC-Net network model proposed in this paper is also a U-Net-based semantic segmentation model that adopts an encoder–decoder architecture. At the encoder stage, HyTC-Net utilizes ConvNext-Tiny, a compact variant of ConvNeXt and a purely convolutional network serving as the backbone of the encoder, which incorporates a Transformer enhancement module and a custom CTA module designed to perceive multi-scale cloud textures. The Transformer component is a lightweight module, and the LM is not a standard Transformer, but rather a type of local convolution with a self-attention mechanism. Two such modules are inserted after each stage to adapt to the convolutions in ConvNext-Tiny, thereby enhancing the capture of high-level features for clouds that span large areas and vary across different scenes and types. The CTA module is a custom attention module that combines channel, texture and frequency attention to extract cloud features at multiple scales. Through the integration of the LM and CTA modules with ConvNext-Tiny, these three components complement one another, enabling the capture of both the features of large cloud areas and the fine textures within the clouds. The bottleneck section utilizes a lightweight ASPP module (L-WASPP), which fuses multi-scale information to capture contextual details and improve cloud segmentation accuracy. The decoder section employs the standard decoding path of U-Net, progressively restoring resolution and fusing features to enhance the accuracy of cloud boundaries. Finally, the DS module utilizes assisted gradient flow to enhance learning in the intermediate layers and generalizes the Dice and IoU loss functions of the OCM model into the Dynamic Tversky (DT) loss function, upgrades the Boundary loss (Boun) function to the Texture-Aware Boundary (TAB) loss function, and introduces a new Consistency Loss function (Con). While the Cross-Entropy (CE) loss remains unchanged, these four loss functions form a new composite loss function combination to enhance cloud detection performance. HyTC-Net also utilizes the AdamW optimizer. According to the paper [21], AdamW is capable of effectively handling the complex textural features found in remote sensing imagery. To prevent overfitting and the loss of boundary details, the model employs a range of advanced training strategies centered on decoupled weight decay, including a single-cycle learning rate and mixed precision, thereby comprehensively enhancing the model’s performance. The overall architecture of the model is shown in Figure 1.

### 2.2. Lightweight Mobile ViT Module

The LM module primarily addresses the challenge of feature recognition in the presence of clouds and bright backgrounds, whilst enhancing the model’s ability to understand the overall scene and improving its capacity to recognize large, continuous cloud formations and thin clouds.

**Figure 2:**
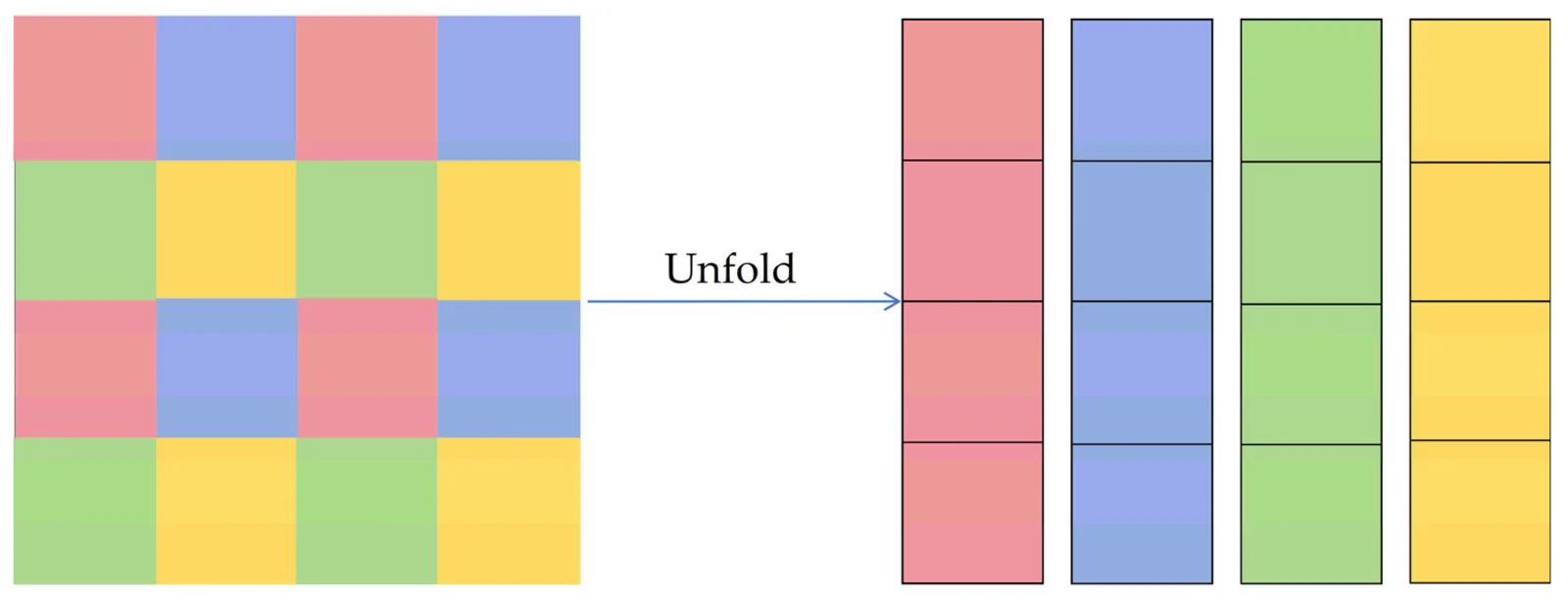
Schematic diagram of the unfold sequence.

The LM module is a lightweight module based on Mobile ViT. Mobile ViT divides an image into a series of non-overlapping image patches, and then uses the multi-head self-attention mechanism within the Transformer to learn the representational relationships between the image patches [22]. This module is a simplified, single-layer Transformer designed for ConvNext-Tiny, which efficiently combines the local feature processing capabilities of convolutions with the global modeling capabilities of the Transformer through an unfold/fold mechanism. The LM module feeds the features into a standard 3 × 3 convolutional layer to identify local features, such as the edge details of clouds, and then into a 1 × 1 pointwise convolution to project the number of input channels to the dimension required by the Transformer. The Transformer, on the other hand, focuses primarily on the global relationships between clouds and the background, enabling a better understanding of contextual relationships. It overcomes the limitations of ConvNext-Tiny and enhances the detection of both individual clouds and multi-layered cloud formations. It is capable of more clearly distinguishing between the background and clouds, particularly when identifying faint clouds, such as scattered clouds, or in complex backgrounds such as snowy landscapes. Next, as shown in Figure 2, the ‘unfold’ process, which is central to reducing computational load, extracts a sequence by forming a set of vectors through average pooling of elements at the same positions across all tiles. This process preserves information from the larger image while reducing the spatial resolution of the features processed by the Transformer. The resulting sequence is then processed by a lightweight Transformer, with each Transformer layer incorporating a multi-head attention mechanism that concatenates the outputs of multiple self-attention units into multiple subspaces, ensuring that the model can focus on different aspects of multi-path information [23]. The feedforward neural network (FNN) enables the model to acquire a global receptive field, which is then unfolded back to the original grid space. At this stage, each pixel in the feature map contains not only information from its immediate surroundings, but also global information. This module enhances the comprehensive recognition of thin clouds, and the final output is generated through global feature fusion. With this lightweight module, the model can better connect to distant cloud regions during cloud detection, enhancing the completeness of cloud detection and the continuity of boundaries, thereby improving the model’s performance metrics.

The self-attention mechanism in the LM module is a core component of the Transformer layer, enhanced by the multi-head attention mechanism. The model takes a sequence *X* ∈ *R*^(*N*×*d*)^ as input, where *N* is the sequence length and *d* is the dimension of each vector. All three matrices are obtained from the input matrix *x* via different linear projections to yield *Q* = *XW^Q^*, *K* = *XW^k^* and *V* = *XW^V^*. The *Q* matrix, known as the query matrix, serves to determine what information the model wishes to extract; the *i* row vector of the *Q* matrix, *q_i_* acts as the query vector for the *i* token in the input sequence and can take context into account. The *K* matrix is the key matrix, where each row *k_j_* of the *K* matrix represents the key vector for the *j* word, which provides retrieval features and is used to calculate the degree of affinity with *q_i_* to generate the scalar product attention $\mathrm{score}\left(i,j\right)=q_{i}\cdot k_{i}^{T}$ to measure the degree of alignment between *q_i_* and *k_i_* in *d_k_* dimensional space. A higher score indicates a stronger correspondence between the information in the *Q* matrix and the label information provided by the *K* matrix. The *V* matrix is a value matrix containing actual information, where each row *v_j_* represents the semantic information that the *j* word in the sequence should convey, and where *W_q_*, *W_k_* and *W_v_* are three independent, learnable weight matrices that enable the model to better understand the context. The final self-attention formula is given by Equation (1):

(1) $$\mathrm{Attention}\left(Q,K,V\right)=\mathrm{softmax}\left(\frac{QK^{T}}{\sqrt{d_{k}}}\right)V$$

**Figure 3:**
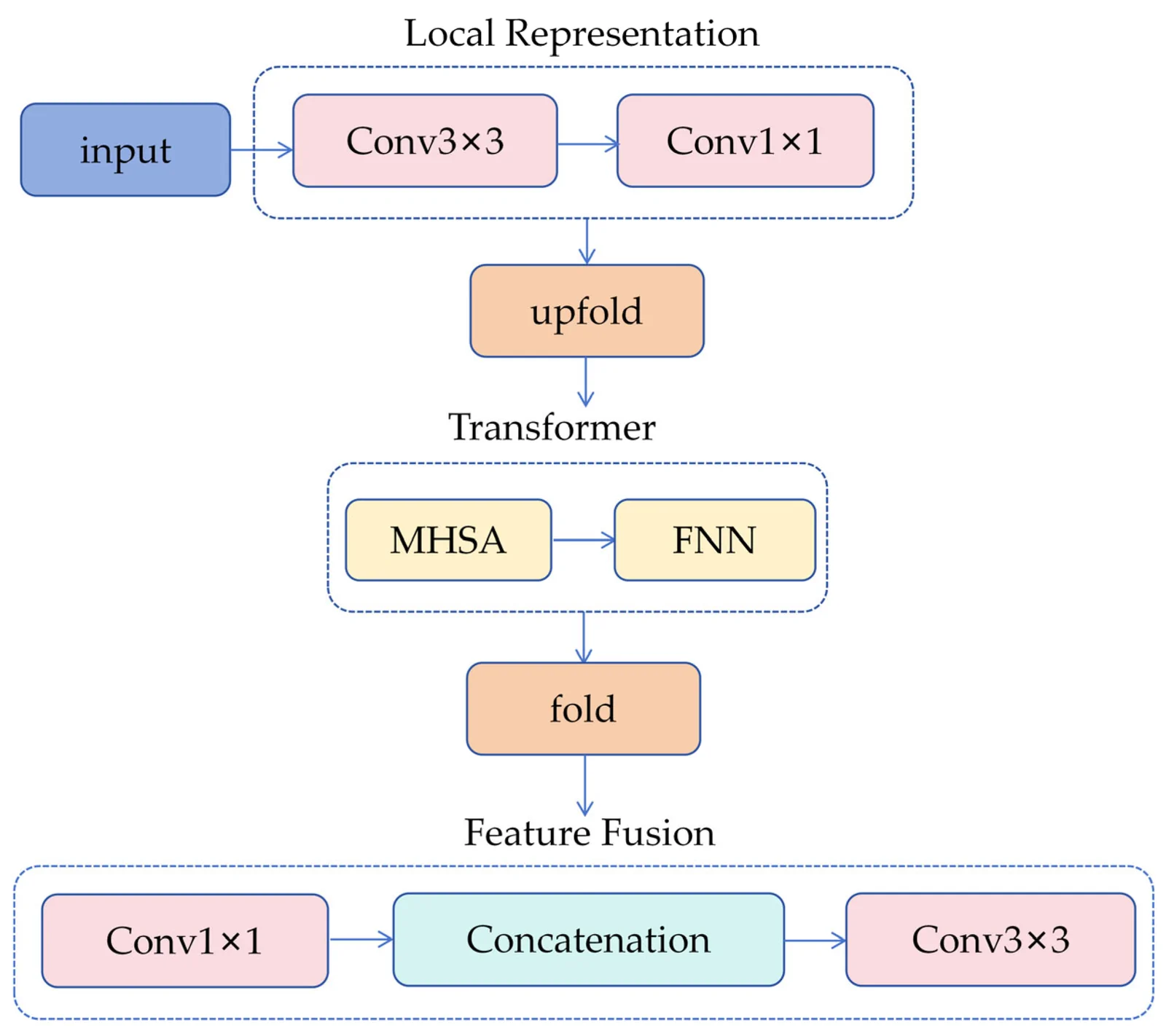
Schematic diagram of the LM module.

As a core component of the Transformer, the feedforward neural network first uses 1 × 1 convolutions to expand the feature channels, thereby enhancing the model’s feature representation capabilities. Subsequently, the GELU activation function is employed to introduce non-linearity, thereby improving the model’s fitting capabilities. The paper [24] mentions the use of the GELU activation function as an alternative to ReLU. ReLU truncates negative inputs to zero, which may result in the loss of feature information in areas of low intensity, such as the edges of thin clouds. In contrast, GELU’s smooth curve preserves the gradients of negative inputs, while its intrinsic gating mechanism also helps to suppress bright background noise. Finally, a dimension reduction layer restores the features to their original dimensions. The specific mathematical expression for the feedforward neural network is given in Equation (2):

*FNN*(*x*) = *σ*(*xW*_1_ + *b*_1_)*W*_2_ + *b*_2_ (2)

where *W*_1_ and *b*_1_ are the weights and biases for the first layer of dimensionality increase, while *W*_2_ and *b*_2_ are the weights and biases for the second layer of dimensionality reduction; a represents the GELU activation function. The overall structure of the LM module is shown in Figure 3 below.

### 2.3. CloudTextureAttention Module

The CTA module combines channel attention, multi-scale texture attention, and a frequency-related branch (F-branch) based on local spatial statistics. It is designed to improve feature representation for cloud detection in high-resolution imagery, particularly in scenes containing thin clouds, fragmented clouds, and bright backgrounds such as snow. In these scenes, spectral and brightness information alone may provide insufficient discrimination. CTA therefore integrates channel dependencies, spatial texture patterns at different scales, and local feature variations to provide complementary information for subsequent segmentation.

Unlike traditional attention mechanisms, it incorporates specific components for texture and frequency features, enabling the characterization of local spatial variations through variance-based statistical descriptors. This allows the model to separately extract and recognize distinct features when processing complex scenes. Channel attention draws on the concept of the SE module, which adaptively recalibrates channel feature responses by explicitly modeling inter-channel dependencies [25]. The dimension reduction ratio is used to reduce the number of channels to minimize the computational load; in cloud imagery, certain channels emphasize the color and texture of the clouds while suppressing irrelevant channels. Through adaptive average pooling compression, a multilayer perceptron (MLP) computes channel weights; the first layer reduces the dimensionality, while the second layer restores it, with ReLU and Sigmoid activation functions jointly generating the channel weights. Specifically, within the general architectural paradigm of MetaFormer, this is used to blend information across channel dimensions and perform non-linear feature transformations [26]. With regard to multi-scale texture attention, the approach primarily captures the various features of clouds by employing multiple deep separable convolutions at different scales, with multi-scale convolutional kernels capturing cloud textures of varying sizes. Convolution is performed separately for each channel, with each convolution kernel processing only a single channel, thereby reducing the number of parameters while maintaining channel independence. The use of convolution kernels of different sizes—namely 3 × 3, 5 × 5 and 1 × 1—enables the capture of texture features at various scales; smaller kernels focus more on local details, while larger kernels focus more on the overall texture patterns of the clouds. This is followed by a forced initialization stage, which acts as an averaging filter. The core principle involves assigning a value to the central pixel by averaging the values of its neighbors within the convolution kernel’s range, as shown in Equation (3):

(3) Outputi,j=1k×k∑m=−k2k2∑n=−k2k2Inputi+m,j+n

In this context, if k is defined as the size of the convolution kernel, then each element is initialized to 1/(*k* × *k*). In a two-dimensional image, this corresponds to the output pixel value at position (*i*,*j*). The primary function of the averaging filter is to smooth the initial features, stabilize the training process, and prevent gradient explosion or vanishing gradients caused by excessively large weights.

Thin clouds and the background may have low local contrast and be obscured by background texture; consequently, it may be difficult to distinguish them based solely on appearance or features at a single scale. To complement the multi-scale convolutional features, the frequency-related branch of CTA uses local statistics to characterize spatial variations in feature maps. Although a global Fourier transform provides an explicit frequency representation, its coefficients do not directly localize these variations within the image. The F-branch instead computes local variance over sliding windows, producing a spatially aligned descriptor of local texture variations without explicitly performing a frequency transform.

The F-branch computes local statistical descriptors directly in the spatial domain. For a feature map *X*, the local mean is obtained by average pooling, and the local variance is calculated as the local mean of squared responses minus the square of the local mean. Both operations can be implemented efficiently using local average pooling with identical window and padding settings. The local variance therefore provides a spatially aligned measure of feature fluctuations within each neighborhood. It may respond to cloud boundaries, heterogeneous cloud structures, background textures, or noise, and is consequently used as a complementary descriptor, rather than as a definitive cloud–background classifier. The local variance is defined in Equation (4).

(4) $$E_{c}\left(i,j\right)=\frac{1}{k\times k}\sum_{\left(m,n\right)\in \Omega \left(i,j\right)}X_{c}{\left(m,n\right)}^{2}-{\left(\frac{1}{k\times k}\sum_{\left(m,n\right)\in \Omega \left(i,j\right)}X_{c}\left(m,n\right)\right)}^{2}$$

where *E_c_*(*i*,*j*) denotes the local variance at position (*i*,*j*) in channel *c*, *X_c_*(*m*,*n*) denotes the corresponding feature value at position (*m*,*n*), and Ω(*i*.*j*) denotes a *k* × *k* local window centered at (*i*,*j*).

To clarify the relationship between local variance and frequency-related information, consider the two-dimensional discrete Fourier transform (DFT) of a local window. Subtracting the local mean removes the DC component, while the local variance measures the average squared magnitude of the remaining fluctuations. According to Parseval’s theorem, this quantity is proportional to the aggregate energy of the non-DC Fourier coefficients. Under the convention of an unnormalized forward DFT, Equation (4) can therefore be expressed as Equation (5). Here, the proportionality factor is determined by the DFT normalization and the number of samples in the local window. Equation (5) provides a mathematical interpretation of the local variance as an aggregate non-DC energy measure. However, the F-branch does not explicitly perform a Fourier transform, select individual frequency bands, or distinguish between low- and high-frequency components. The relationship is used only to interpret the spatial local-variance descriptor.

(5) $$E_{c}\left(i,j\right)=\frac{1}{k^{4}}{\sum_{\begin{array}{l} 0\leq u,v<k\\ \left(u,v\right)\neq \left(0,0\right) \end{array}}\left|{\hat{X_{c}}}^{\left(i,j\right)}\left(u,v\right)\right|}^{2}$$

where ${\hat{X_{c}}}^{\left(i,j\right)}\left(u,v\right)$ denotes the DFT coefficient at frequency index (*u*,*v*) of the local window centered at (*i*,*j*) in channel *c*, and (*u*,*v*) = (0,0) corresponds to the DC component.

**Figure 4:**
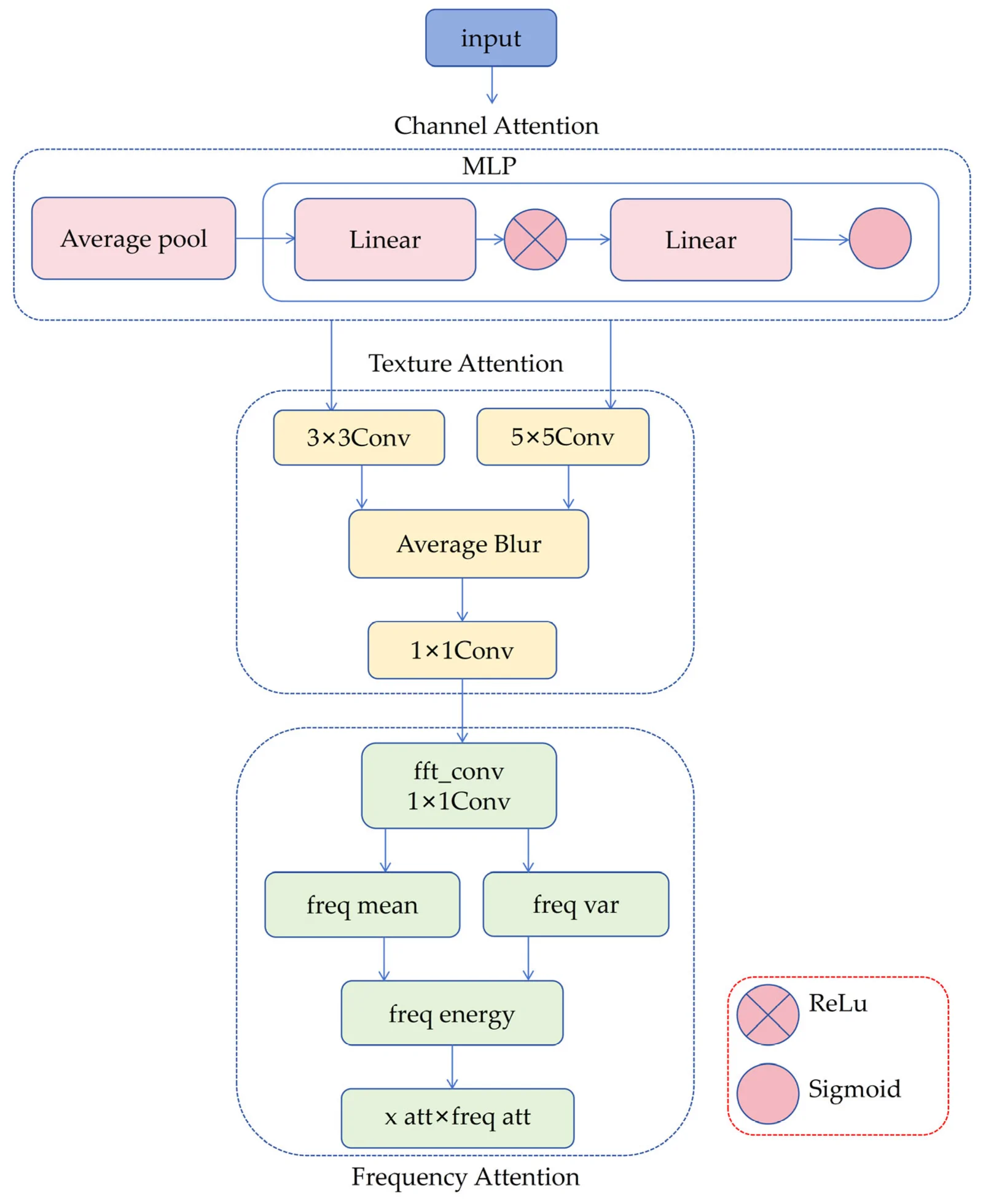
CTA module block diagram.

As discussed in a previous article, the AMFF network utilizes attention layers that are aware of frequency to emphasize relevant and important frequencies within the multiband embedding matrix, thereby capturing frequent patterns in sequence recommendation [27]. The CTA module is inserted after the four encoder stages to process features at different spatial resolutions and semantic depths. The same local statistical operation can consequently describe variations at different effective receptive-field scales. Channel attention adjusts the relative importance of feature channels, multi-scale texture attention captures neighborhood patterns, and the F-branch supplies a complementary descriptor of local feature fluctuations. Figure 4 shows the architectural diagram following the integration of this module.

**Figure 5:**
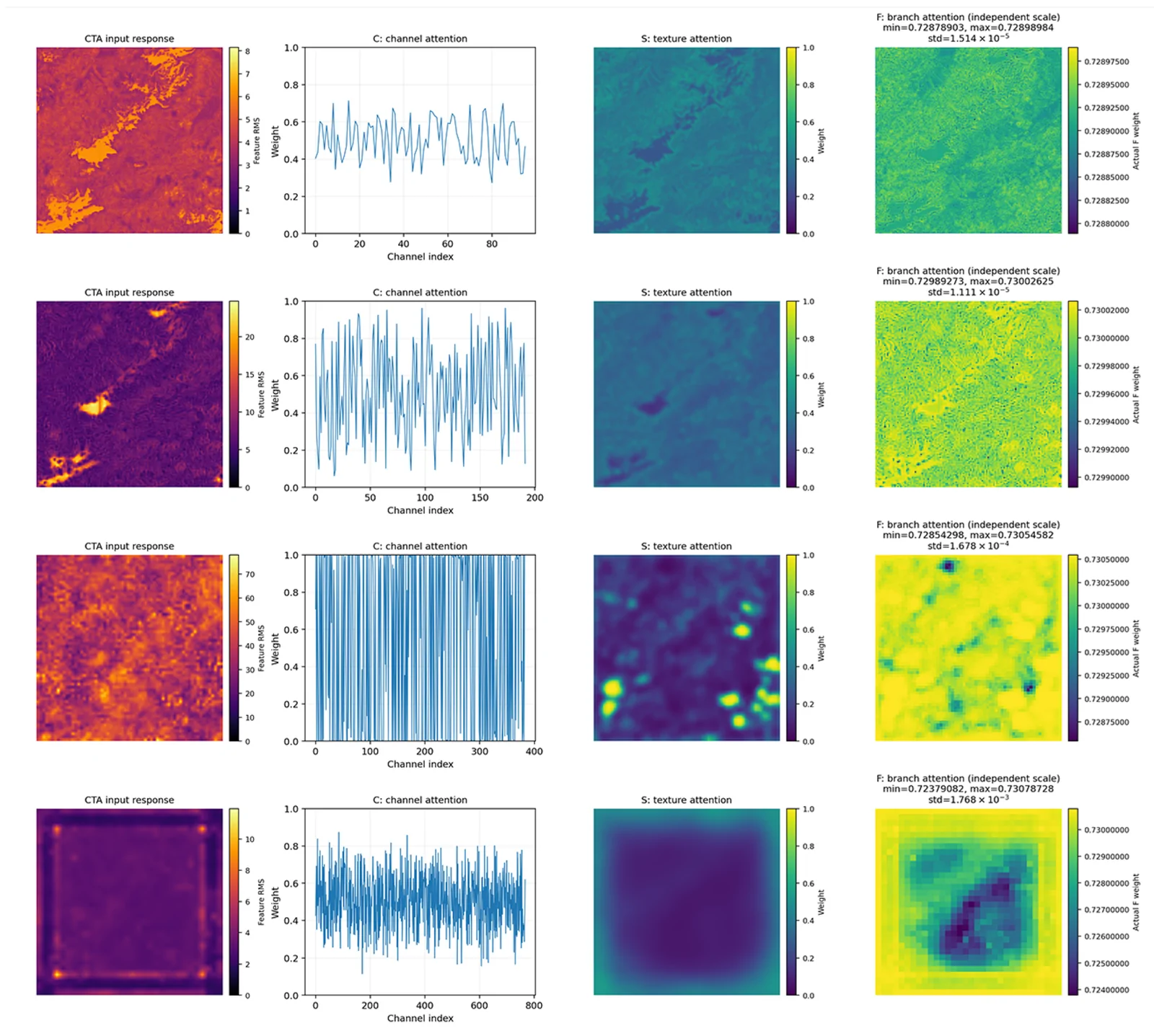
Visualization of internal attention responses of CTA at four feature stages.

**Figure 6:**
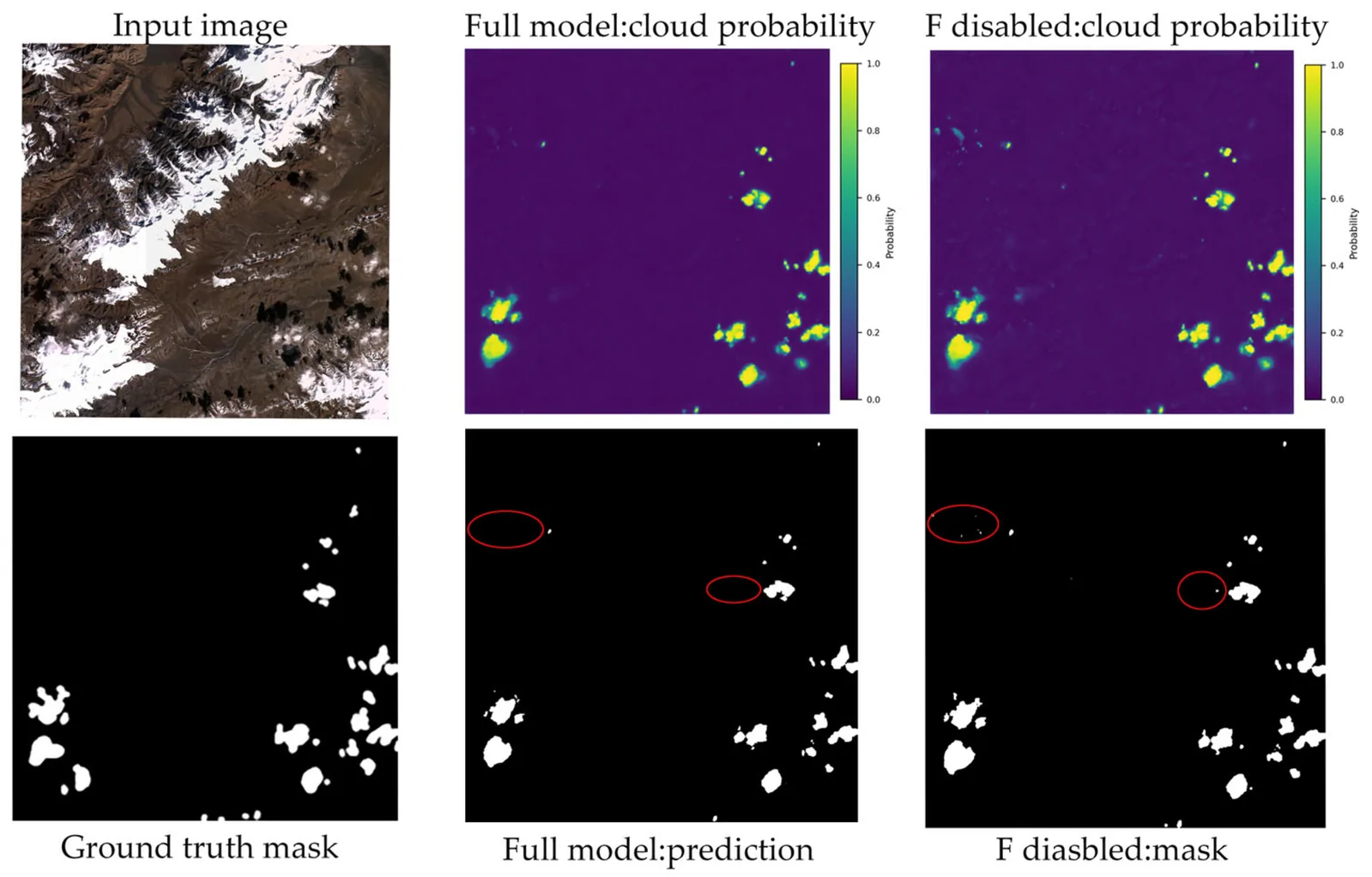
Comparison of cloud detection results with and without F-branch modulation during inference.

To examine the behavior of CTA, we visualize the internal attention responses at the four encoder stages in Figure 5. The F-branch maps represent spatial modulation weights derived from local statistical descriptors; they are neither cloud probabilities nor Fourier spectra. We additionally compare the full model with a configuration in which F-branch modulation is disabled during inference, as shown in Figure 6.

Rows correspond to Stages 1–4. From left to right, the columns show the root-mean-square (RMS) magnitude of the CTA input features, channel attention weights of the C-branch, spatial attention maps of the S-branch, and attention maps of the F-branch. The C-branch plots show attention weights against channel indices. The F-branch maps use independent color scales, with their minimum, maximum, and standard deviation reported above each map.

Figure 5 presents the internal attention responses of CTA at four feature stages. The C-branch assigns different weights to individual channels, with pronounced channel-wise selectivity at Stage 3, where many weights approach either 0 or 1. The S-branch exhibits spatially varying responses: relatively localized high-response regions appear at Stage 3, whereas a smoother, broader pattern is observed at Stage 4. The F-branch weights remain close to 0.73 across all stages, but their spatial variation differs with feature depth. The standard deviations are approximately 1.51 × 10^−5^, 1.11 × 10^−5^, 1.68 × 10^−4^, and 1.77 × 10^−3^ for Stages 1–4, respectively. Thus, the first two stages exhibit nearly uniform modulation, while the deeper stages show more noticeable spatial variation. These observations characterize the distinct weighting patterns of the three branches.

The top row shows the input image and cloud probability maps produced by the full model and the model with F-branch modulation disabled. The bottom row shows the ground-truth mask and the corresponding predicted masks. Both probability maps use the same color scale from 0 to 1. Red ellipses highlight locations where disabling F-branch modulation introduces additional isolated cloud predictions. The trained model parameters remain unchanged, and no retraining is performed.

Figure 6 examines the influence of F-branch modulation on the final cloud predictions by disabling it during inference while retaining the trained model parameters. Both configurations preserve the main predicted cloud regions, indicating that the overall segmentation pattern remains broadly similar in this example. Nevertheless, disabling F-branch modulation produces additional scattered responses in the probability map and isolated positive predictions within the highlighted regions. These isolated predictions are absent from the corresponding ground-truth locations and therefore represent local false positives. In comparison, the full model yields fewer such responses in these regions. This example suggests that F-branch modulation contributes to suppressing some spurious background responses. The comparison assesses the sensitivity of the trained model to F-branch modulation.

### 2.4. LightWeightASPP Module

The L-WASPP module, a lightweight version of the traditional ASPP module, was first used in MobileNetV3 and related work as a highly efficient segmentation decoder [28] designed to maintain the original semantic segmentation accuracy. This module primarily addresses the issue that, as satellite imagery contains both large and small patches of cloud, a fixed field of view struggles to handle this situation comprehensively; furthermore, during the sampling process in the decoder, small details are easily lost, resulting in blurred cloud boundaries in the final prediction.

**Figure 7:**
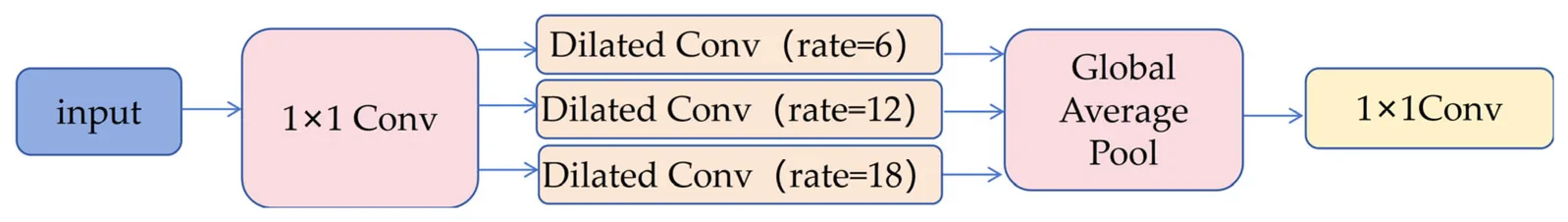
LightWeightASPP module architectural diagram.

By employing multiple parallel convolutions, the model can capture contextual information at different scales while maintaining the resolution of the feature maps. This enhances the model’s ability to extract global cloud features. One of these is the 1 × 1 convolutional layer in the main branch; however, as the receptive field of a 1 × 1 convolution is very small, it primarily focuses on local features and is computationally lightweight. It can also be regarded as a linear combination across the channel dimension, which facilitates information exchange between different channels. The core of dilated convolution lies in the dilation and padding parameters. It utilizes a 3 × 3 convolution kernel, which is more lightweight than the traditional 5 × 5 and 7 × 7 kernels. Dilation determines the sampling interval of the convolution kernel, thereby expanding the receptive field and placing greater emphasis on capturing global features. Before dilating the receptive field, the value of the padding must be determined, as this value should be equal to the dilation value to ensure that the spatial resolution remains unchanged. This paper employs three types of dilated convolution, with dilation rates of 6, 12 and 18. The smaller the dilation rate, the smaller the coverage area compared to larger dilation rates, which allows for a focused examination of local edge details in patchy cloud formations, while the module continues to focus on global contextual features. Subsequent global average pooling effectively reduces false positives caused by bright features such as snow cover, based on the context, while also extracting global image features. As there are five branches, a 1 × 1 convolutional layer is required to perform overall feature fusion, merging the five channels together to include feature information from different receptive fields. In response to the multi-scale variations in clouds, this module, having fused features from different receptive fields, not only focuses on details, but also considers the global picture, enabling it to handle clouds of various sizes; furthermore, the use of a blended dilation rate helps to eliminate the grid effects caused by high dilation rates. Although a large dilation factor can help the model understand context, it can also result in significant information gaps; combining it with a small dilation factor can fill these gaps, enabling more comprehensive feature capture. The architecture of this module is shown in Figure 7.

### 2.5. Robust Hybrid Multi-Objective Loss Function

As traditional loss functions still have certain limitations when handling high-precision segmentation tasks, it is difficult to account for both complex textural details and global structure simultaneously. Therefore, this paper proposes a robust hybrid multi-objective loss strategy; the original model already combines four loss functions—CE, Dice, IoU and Boundary—aiming to achieve multi-dimensional joint supervision. The composite loss function proposed in this paper enables the simultaneous optimization of pixel-level classification accuracy and the identification of challenging samples, while reducing the misclassification rate of highlights and enhancing the model’s robustness in handling clouds under varying lighting conditions and morphologies. Consequently, it significantly improves the model’s segmentation accuracy in complex scenes, as well as the stability of its segmentation performance. The original model utilized a combination of four traditional loss functions. In the experiments conducted in this paper, the CE loss function remains unchanged, while the Dice and IoU loss functions are replaced by the DT loss function. The core objective is to severely penalize false positives, such as mistaking snow for clouds, by dynamically adjusting the two hyperparameters alpha and beta. The TAB is an upgraded version of the original Boun. It utilizes the texture of the input boundaries to impose heavier penalties in areas of smooth texture and lighter penalties in regions with complex texture boundaries. Furthermore, to prevent the model from predicting a continuous thin cloud as a series of discontinuities, or from erroneously filling gaps within the cloud, a new ‘Consistency’ smoothing regularization loss function has been introduced. Its primary function is regularization; by encouraging consistent predictions in response to perturbed inputs, it smooths the final segmentation results to remove minor noise and artifacts, thereby enhancing the model’s segmentation accuracy. To address this issue, this paper employs a combination of four loss function components, as shown in Equation (6):

*L_total_* = *λ*_1_*L_CE_* + *λ*_2_*L_DT_* + *λ*_3_*L_TAB_* + *λ*_4_*L_Consistency_* (6)

here, *λ_n_* represents the weight coefficients assigned to the four loss functions; *L_CE_* represents the cross-entropy loss function; *L_DT_* represents the DT loss function; *L_TAB_* represents the texture-boundary-aware loss function; and *L_Consistency_* represents the smoothing regularization loss function.

The CE cross-entropy loss function is the de facto standard objective function in semantic segmentation tasks; its theoretical basis stems from maximum likelihood estimation in information theory. As noted in the paper [29] on fully convolutional networks, extending the traditional image-level classification loss to the spatial dimension, by computing an independent logical loss at each spatial location and averaging these, can effectively drive the network to perform end-to-end dense predictions, as shown in Equation (7):

(7) $$L_{\mathrm{CE}}=-\frac{1}{N}\sum_{i=1}^{N}y_{i}\log\left({\hat{y}}_{i}\right)+\left(1-y_{i}\right)\log\left(1-{\hat{y}}_{i}\right)$$

here, *y_i_* represents the true label of the *i* pixel, and *y_i_* represents the probability predicted by the model that the pixel is a cloud. In deep learning, the CE loss function is used to measure the discrepancy between the predicted probability distribution and the true label distribution, that is, the Kullback–Leibler divergence, which quantifies the difference between the two distributions. As the target class accounts for a small proportion of the total samples, this paper sets the class weight for the background to 1.0 and that for the target cloud to 4.0 to address the issue of class imbalance.

The DT loss function is an improved version of the Tversky loss function, designed to handle the class imbalance commonly encountered in image segmentation with greater flexibility. While traditional loss functions employ fixed hyperparameters, the DT loss function introduces two weight parameters, *α* and *β*, to control the severity of penalties for false positives and false negatives, as shown in Equation (8):

*Tversky*(*α*, *β*) = *TP*/(*TP* + *αFP* + *βFN*) (8)

when *α* < *β* is set to a higher value, the function places greater emphasis on reducing false negatives, that is, it prioritizes recall; when *α*, the focus shifts to reducing false positives, emphasizing precision. The loss function is typically defined as Equation (9):

*L_Tversky_* = 1 − *Tversky*(*α*, *β*) (9)

The term ‘dynamic’ refers to the fact that the parameters *α* and *β* in the formula are no longer fixed hyperparameters, but are dynamically adjusted during training according to the criteria, specifically based on class imbalance. The loss function can adjust the values of *α* and *β* in real time based on the ratio of foreground to background pixels in the current batch, ensuring that optimal balance is achieved regardless of the degree of imbalance. Regarding dynamic adjustment based on performance, as training progresses, if the model performs poorly on a particular metric such as recall, the dynamic mechanism will increase the penalty weight for that metric, prompting the model to converge rapidly to a more optimal balance.

TAB is a texture boundary-aware loss function. This is a loss function comprising both Boun and texture loss, designed primarily to address the issues of blurred boundaries and loss of internal texture information; it combines a precise focus on boundaries with the preservation of texture features in internal regions. Traditional loss functions are insensitive to minor boundary shifts; the TAB enhances both boundary sensitivity and texture awareness. Its structure is as shown in the equation *L_TAB_* = *λ*_1_*L_Boundary_* + *λ*_2_*L_Texture_*. Among these, the Boun loss typically uses distance metrics to measure the geometric discrepancy between the predicted boundary and the ground-truth boundary, while texture loss primarily calculates the difference between the predicted segmentation region and the ground-truth region in feature space by utilizing pre-trained networks such as VGG to extract feature maps, and compares the local gradient information or grey-level statistics of the predicted and ground-truth regions to ensure that internal texture details are correctly reconstructed [30]. To ensure that these two enhancement modules—one for texture perception and the other for boundary perception—function effectively, a texture boundary detection loss function has been incorporated to address segmentation tasks involving complex and subtle texture features.

Consistency is referred to as Consistency Loss, also known as consistency regularization. In traditional deep learning, we use only labeled data to compute the loss. However, if we have a large amount of unlabeled data, consistency regularization utilizes this unlabeled data to guide the model in learning more robust features. Consistency Loss is a powerful regularization tool which enhances the model’s stability of model segmentation performance and the quality of its feature representations in situations where data is sparse or labels are incomplete, by forcing the model’s output to remain stable in the face of minor changes to the input. The paper [31] demonstrates that Consistency Loss ensures that all objects within the same set of shapes share consistent semantic segmentation results, even if they have different specific shapes, and forces the model to maintain output stability in the face of input perturbations, as shown in Equation (10):

*L_consistency_* = *Distance*(*P*(*f*(*x*)), *P*(*f*(*x*^′^))) (10)

where *x* is the original input, *x*^′^ is a version of *x* to which some form of disturbance has been applied, such as data augmentation, *p*(⋅) is the probability distribution or prediction output of the model, and *Distance*(⋅,⋅) is a distance function that measures the difference between the two outputs, used to quantify the differences between different input views or predictions at different times.

The four component loss functions are combined; the CE, cross-entropy loss function, is the primary loss function for the imbalanced class and is therefore assigned a value of 0.3. As the dataset contains a high proportion of challenging samples, such as fragmented clouds, thin clouds, and highlights, DT serves as the foundation, ensuring that pixels belonging to the background and target cloud classes are classified correctly, while capturing the main subject of the segmentation to prevent confusion between clouds and snow; consequently, it is assigned a dominant weight of 0.55. Replacing the Dice and IoU loss functions in the original model with this approach enables more precise segmentation. TAB Loss primarily focuses on boundary segmentation tasks, utilizing the image’s texture information to guide the model’s learning of boundaries. By replacing the original model’s Boun Loss, it enables more precise handling of complex boundaries; consequently, it also occupies a relatively dominant position, with a weighting of 0.1; Consistency Loss is used to smooth the final segmentation results by encouraging consistent predictions for perturbed inputs, thereby removing minor noise and artifacts. It enhances the model’s stability of model segmentation performance, while its role in regularization is supplementary; consequently, it is assigned the lowest weight of 0.05. This combined design ensures that the model performs exceptionally well across five key aspects: classification accuracy, focus on difficult samples, metric optimization, clear boundaries, and gradient stability.

### 2.6. Deep Supervision Module

The paper [32] points out that, as the depth of a network increases, gradients gradually vanish during the backpropagation process, making it difficult to update the parameters of shallow layers. To address the issue of gradient vanishing during the training of deep convolutional neural networks, and to enhance the model’s ability to extract multi-scale cloud features, we have introduced a deep supervision mechanism, DS, at the decoder stage. By introducing branches at the intermediate layers of the decoder to directly compute the loss function, we ensure that both the deep and intermediate layers possess semantic segmentation capabilities. Given that the network architecture proposed in this paper features a deep encoder–decoder structure, gradients are prone to attenuation during backpropagation as the network depth increases. By introducing branches at the intermediate layers of the decoder, this paper introduces three branches at the three intermediate layers of the decoder; each branch contains a 1 × 1 convolutional layer that compresses different high-dimension feature channels into binary classification channels, thereby shortening the gradient propagation path and accelerating model convergence. During the training phase, these low-resolution predicted images are upsampled to the original image size via bilinear interpolation and used to compute an auxiliary loss with the ground-truth mask, as shown in Equation (11):

(11) $$L_{\mathrm{total}}=L_{\mathrm{final}}+\sum_{i-1}^{3}\lambda_{i}L_{\mathrm{aux}\_i}$$

where *L_final_* refers to the main layer output branch, while *L_aux_*__*i*_ denotes the auxiliary branch of the *i* layer; in the method adopted in this paper, a weight of 0.4 is assigned to both branches to balance them. This component is activated only during the training phase; compared with the original model without DS, DS provides additional supervisory signals to intermediate decoder features during training. During the upsampling process, this also prevents the misclassification of fragmented and thin clouds that can occur when features are directly restored, thereby enhancing the model’s ability to perceive features across multiple scales; the DS module is also comprehensive in terms of detail. As the auxiliary head at the shallowest layer receives supervisory signals, it learns more precise edge features, and the final output layer fuses these features that have undergone strong supervision. At the same time, DS acts as a form of regularization, primarily preventing the model from overfitting during training, enhancing its robustness.

## 3. Results

### 3.1. Experiments and Results Analysis

#### Datasets and Experimental Environment

**Figure 8:**
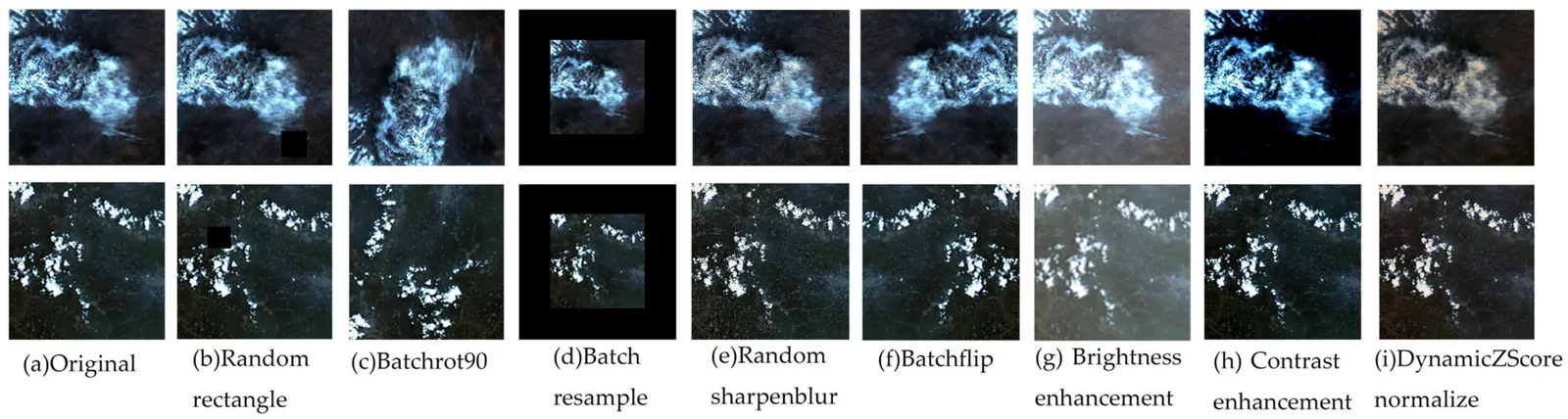
Examples of data augmentation: (**a**) Original; (**b**) Random rectangular; (**c**) BatchRot90; (**d**) Batch resample; (**e**) Ran-dom sharpening and blurring; (**f**) Batchflip; (**g**) Brightness enhancement; (**h**) Contrast enhancement; and (**i**) Dynamic ZScore normalization.

The dataset used in this paper consists of satellite images 01 and 02 from the ZY-3 satellite; these images have been processed and adjusted to a size of 1024 pixels × 1024 pixels. Data augmentation techniques such as random occlusion, 90-degree rotation, scaling, horizontal flipping, sharpening, brightness enhancement, contrast enhancement and dynamic Z-score standardization were applied, as shown in Figure 8. The volume of data was increased to improve the stability of the model’s segmentation results, and a custom dataset was created, categorized by difficulty level into thick clouds, scattered clouds, thin clouds and clouds against a snowy background. Reference cloud masks were generated using a semi-automatic annotation procedure combining manual delineation, intensity-based thresholding, and manual refinement in Adobe Photoshop. Candidate cloud regions were first manually delineated, and thresholding was subsequently applied within these regions to generate preliminary masks. For the 8-bit images used during annotation, threshold values were adjusted according to cloud appearance, typically ranging from 200 to 240 for thick clouds, 140 to 190 for thin clouds, and 160 to 210 for small, scattered clouds. These ranges served as initial settings and were adjusted as necessary to accommodate variations in image brightness and cloud characteristics. Because the ZY-3 multispectral imagery lacks a shortwave infrared band, a normalized red–near-infrared spectral contrast was used as an auxiliary cue for interpreting ambiguous cloud–snow regions. For the ZY-3 multispectral imagery, a normalized red–near-infrared spectral contrast was calculated as *C* = (*ρ_red_* − *ρ_NIR_*)/(*ρ_red_* + *ρ_NIR_* + *ε*), where *ρ_red_* and *ρ_NIR_* denote the red- and near-infrared-band reflectance, respectively, and *ε* is a small positive constant used to avoid division by zero. This spectral contrast was used only as an auxiliary cue during manual mask refinement, together with visual interpretation and spatial context, rather than as a standalone criterion for cloud–snow discrimination. The index was considered alongside the spatial context and visual characteristics of the original imagery. The preliminary masks were subsequently inspected and manually refined, with particular attention to thin cloud boundaries, small and scattered clouds, and regions containing both clouds and snow. Manual corrections were applied to remove incorrectly labeled background regions and recover omitted cloud pixels, yielding the final reference cloud masks.

**Figure 9:**
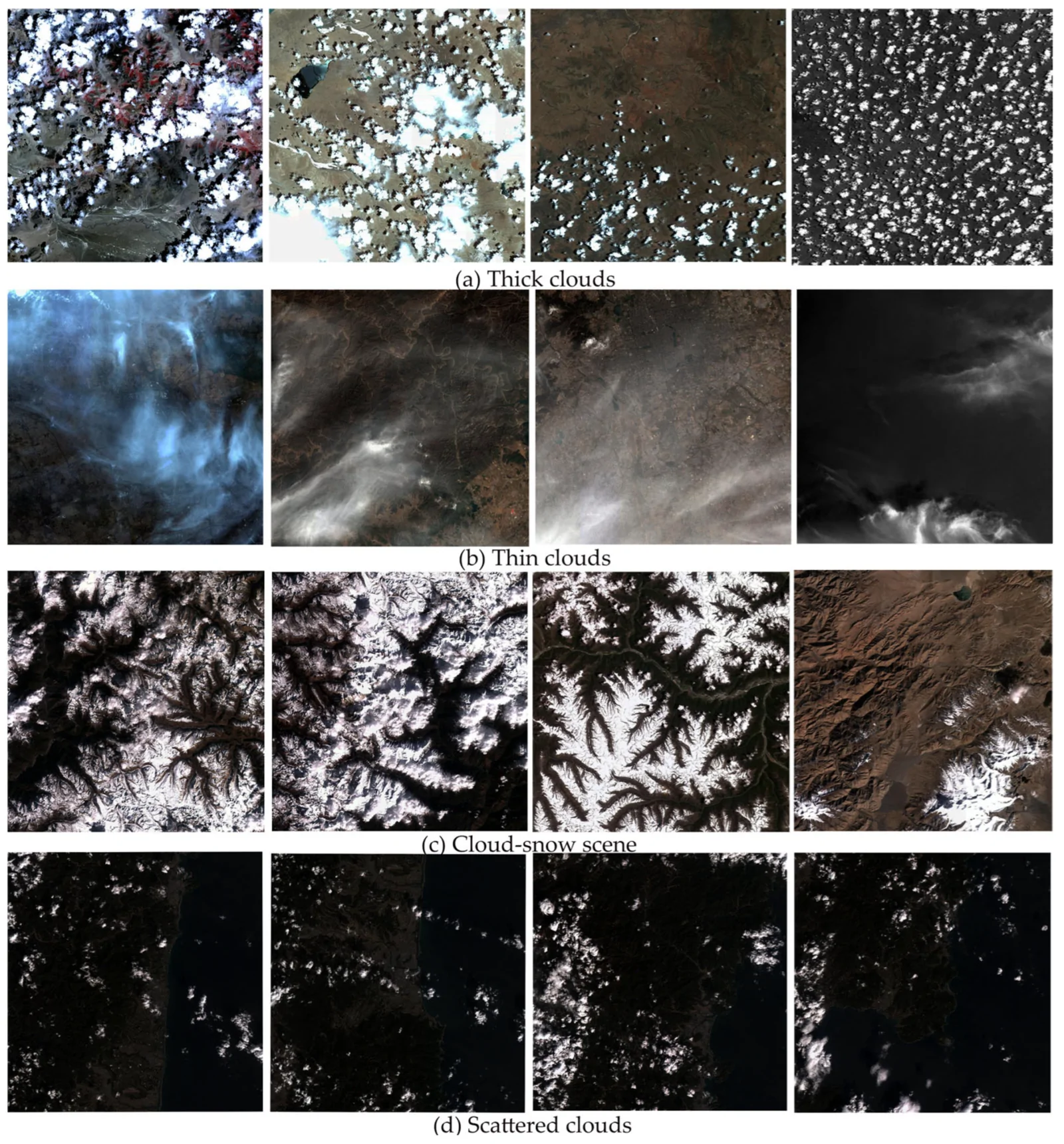
Satellite imagery from the ZY-3 satellite: (**a**) thick clouds; (**b**) thin clouds; (**c**) cloud–snow scenes; and (**d**) scattered clouds.

The ZY-3 satellite is China’s first civilian high-resolution satellite designed specifically for the acquisition of stereo imagery along its orbit. It has a spatial resolution of 2.1 m at the zenith and a spatial resolution of 3.6 m at both the forward and backward viewing angles. The forward-looking and backward-looking cameras have angles of incidence of ±22° relative to the zenith camera, enabling the simultaneous acquisition of multi-view panchromatic imagery; this unique advantage makes it particularly well-suited to the extraction of vertical features on the Earth’s surface [33]. The ZY-3 satellite features four spectral bands: red, green, blue and near-infrared. As it has only four bands—lacking the short-wave infrared and thermal infrared bands—it becomes more difficult to extract clouds from images where both clouds and highly reflective ground features, such as snow-covered areas, are present. Furthermore, due to the high resolution of the imagery, many details are magnified; the higher the clarity, the more ground features exhibit spectral characteristics like those of clouds, leading to misclassifications. The satellite imagery is shown in Figure 9.

As the resolution of the raw imagery is inconsistent and the model is designed for batch processing, bilinear interpolation is used to upscale the raw imagery to a resolution of 1024 × 1024. This not only facilitates batch processing of the model, but also ensures that the decoder and encoder match the dimensions of the feature maps. The mask was processed using nearest-neighbor interpolation. As a value of 0 in the mask represents the background and 1 represents clouds, and to avoid fractional values at class boundaries, the nearest-neighbor interpolation method was adopted to ensure that labels are exclusively 0 or 1. In this experiment, a total of 1833 original images were collected. The images were grouped by their source scenes, and the resulting groups were randomly assigned to the training, validation, and test sets to achieve an approximate image count ratio of 8:1:1. The resulting training, validation, and test sets contained 1467, 183, and 183 original images, respectively. All images originating from the same source scene were assigned exclusively to a single subset. After partitioning, eight data augmentation methods were applied exclusively to the training set, yielding 13,203 training images, including the original training images. The validation and test sets were retained without augmentation.

The data for this experiment were primarily selected from challenging images with complex backgrounds, The selected regions are in northwestern and southwestern China, where cloud cover ranges from 20% to 40%, and include arid land, as well as snow- and ice-covered areas. These regions generally have high reflectance values that are extremely similar to those of cloud cover. This dataset contains significant reflectance interference, enabling the model to perform well even under the constraints of the sensor’s spectral bands. Images with water bodies in the background were selected. Water bodies have extremely low reflectance, creating a sharp contrast with the highly reflective land background. The diversity of the imagery enhances the stability of the model’s segmentation.

The experimental parameters for this paper were set as follows: to address the mismatch between the distribution of pre-trained weights and that of randomly initialized weights, a two-stage transfer learning optimization strategy was adopted, comprising a freezing stage and a thawing stage [34]. By freezing the parameters of the backbone network, we forcibly blocked the backpropagation of gradients to the deep feature extractors. During this stage, only the stable feature maps output by the backbone network were utilized to optimize the random parameters of the decoder, enabling it to converge rapidly to a relatively reasonable parameter space, thereby achieving gradient stabilization. In this experiment, a constant learning rate of 8 × 10^−5^ was set for the freezing phase, comprising a total of six rounds of freezing training, followed by 100 rounds of thawing training, during which the learning rate was linearly interpolated between 1 × 10^−6^ and 8 × 10^−5^. The batch size was set to 8, with a total of 106 iterations. All experiments, including comparative experiments between different models and ablation experiments, used the same training, validation, and test splits, as well as the same evaluation protocol. Each configuration was trained using the same set of five random seeds, and the results are reported as mean ± standard deviation across these runs. For identical configurations appearing in multiple tables, the same experimental results are reported to ensure consistency. The scene-specific results in experiments on the detection of different types of clouds under different models were obtained using the same trained checkpoints as those used in experiments on cloud segmentation using different models, without additional scene-specific training. Each checkpoint was evaluated separately on the corresponding scene subsets of the fixed test set. Results are reported as the mean ± sample standard deviation across the five independent training runs.

The experimental environment is shown in Table 1 below: (a) thick clouds; (b) thin clouds; (c) cloud–snow scenes; and (d) scattered clouds.

### 3.2. Evaluation Criteria

This experiment utilizes a semantic segmentation model, with the primary task being to detect areas containing clouds in remote sensing imagery; there are only two pixel classes: clouds and background. Consequently, the model is evaluated using eight metrics: accuracy, precision, recall, mean Intersection-over-Union (mIoU), F1 score, the number of model parameters, Boundary IoU (BIoU) and Boundary F1 (BF1). Accuracy is the most intuitive metric for assessing a model’s overall classification performance; it is defined as the proportion of correctly classified samples—including both positive and negative examples—within the total sample space. Precision is used to quantify the proportion of samples correctly predicted as positive that are, in fact, true positives. Recall is used to measure the proportion of true positive samples correctly identified by the model. mIoU is the recognized gold standard metric for semantic segmentation tasks; it calculates the ratio of the intersection to the union between the predicted mask and the ground-truth mask and takes the average across all classes. The F1 score is the harmonic mean of precision and recall and is the primary metric for evaluating imbalanced classification data. Precision, recall, and F1 score were computed independently for each test image, with cloud pixels designated as the positive class. The resulting image-level metrics were subsequently averaged across the test set, assigning equal weight to each image. Accordingly, the reported F1 score represents the arithmetic mean of the image-level F1 scores, rather than the harmonic mean of the averaged precision and recall. Owing to the nonlinearity of the harmonic mean, these two aggregation procedures are not mathematically equivalent. Consequently, the reported mean F1 score cannot generally be reconstructed directly from the mean precision and recall presented in the tables.

The model’s size is measured using the number of parameters; an excessive number of parameters can lead to overfitting and consume significant computational resources, while too few parameters can result in underfitting. The formulas for the various evaluation indicators are shown in Equations (12)–(17).

To quantitatively evaluate the model’s performance under conditions of thin clouds, patchy clouds, and blurred boundaries, we have further introduced fine-grained evaluation metrics: BIoU and BF1. Traditional IoU is biased towards interior pixels. Boundary IoU focuses exclusively on the segmentation quality of the contour regions. It is calculated by extracting the boundary regions of both the predicted mask and the ground-truth mask within a certain pixel distance *d* (set to 3 pixels in this study) and then computing the IoU within these regions. The formulas for BIoU and BF1 are shown in Equations (18) and (19):

(12) $$\mathrm{Accuracy}=\frac{\mathrm{TP}+\mathrm{TN}}{\mathrm{TP}+\mathrm{TN}+\mathrm{FP}+\mathrm{FN}}$$

(13) $$\mathrm{precision}=\frac{\mathrm{TP}}{\mathrm{TP}+\mathrm{FP}}$$

(14) $$\mathrm{recall}=\frac{\mathrm{TP}}{\mathrm{TP}+\mathrm{FN}}$$

(15) $$\mathrm{Io}U_{i}=\frac{TP_{i}}{TP_{i}+FP_{i}+FN_{i}}$$

(16) $$\mathrm{MIoU}=\frac{1}{k+1}\sum_{i=0}^{k}\mathrm{Io}U_{i}$$

(17) $$F_{1}=\frac{2\times \mathrm{TP}}{2\times \mathrm{TP}+\mathrm{FP}+\mathrm{FN}}$$

(18) $$\mathrm{BIoU}=\frac{\left|\left(P_{d}\cap G_{d}\right)\right|}{\left|\left(P_{d}\cup G_{d}\right)\right|}$$

(19) $$\mathrm{BF}1=\frac{2\times {\mathrm{Precision}}_{d}\times {\mathrm{Recall}}_{d}}{{\mathrm{Precision}}_{d}+{\mathrm{Recall}}_{d}}$$

where *TP* stands for true positive, *TN* for true negative, *FN* for false negative and *FP* for false positive; *P_d_* and *G_d_* represent the boundary regions of the prediction and ground truth; *Precision_d_* is the precision of the bound; and *Recall_d_* is the recall of the bound. The numerator of the *BF*1 formula represents the number of boundary points that have been correctly matched, and the denominator represents the total number of boundary points to be taken into account.

### 3.3. Comparative Experiments

#### 3.3.1. Comparative Experiments Between Different Models

In order to further verify that the performance of the improved network model, HyTC-Net, proposed in this paper, has indeed been enhanced, and to demonstrate the feasibility and effectiveness of the improved model, comparative experiments were conducted using the method described in this paper against eight comparison models: OCM, Unet, DeepLabV3+, TransUnet, HRNet, SwinUnet, D-LinkNet, and Swin-UperNet. The results are shown in Table 2.

For the comparative experiments, two types of clouds were selected: thick clouds and scattered clouds. Thick clouds are relatively easier to segment, while scattered clouds are relatively more difficult to segment. The resolution of the images used in the comparative experiments was 1024 pixels × 1024 pixels.

**Figure 10:**
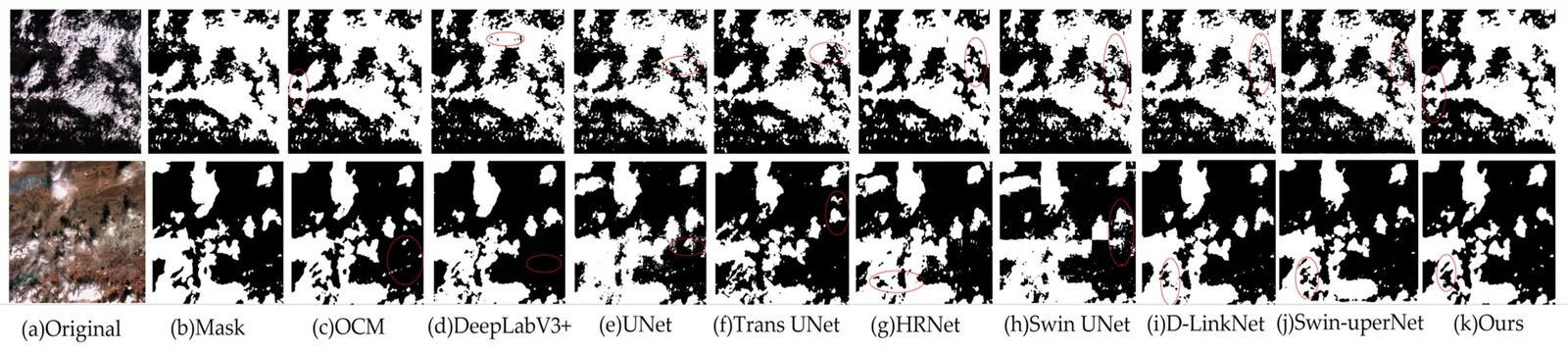
Visual comparison of cloud segmentation results obtained using different models: (**a**) original; (**b**) Mask; (**c**) OCM; (**d**) DeepLabV3+; (**e**) UNet; (**f**) TransUNet; (**g**) HRNet; (**h**) Swin UNet; (**i**) D-LinkNet; (**j**) Swin-uperNet; and (**k**) Ours.

In Figure 10, the dense cloud type demonstrates more clearly that the baseline model, OCM, predicts the background area on the left-hand side of the image as cloud. Other comparison models also exhibit missed detections when detecting dense clouds, whereas the model proposed in this paper has fewer missed and false detections compared to the other models. In the detection of patchy clouds, the baseline model OCM incorrectly predicted the background in the patchy cloud region on the right as cloud, and there were also missed detections in the cloud region on the left. The other comparison models missed all or most of the patchy clouds on the right, and in the thick cloud region on the left, most of the clouds were incorrectly classified as background. As the OCM’s encoder learns features through convolutional layers without any specific enhancement processing, in cases of patchy cloud cover or areas where the transparency of thick clouds is uneven due to high-resolution imagery, the model may classify these sections as background texture or ignore them altogether, leading to missed detections. Although the receptive fields of the U-Net architecture in the OCM model increase as the network deepens, they essentially remain focused on local context. When the model detects large areas of white information, it may, due to the local nature of the receptive fields, mistakenly identify white clouds as large patches of bright background, thereby leading to false positives. As for classical CNN architecture, while the DeepLabV3+ and D-LinkNet models have expanded their receptive fields, they still have limitations, and their dilated convolutions are not sufficiently precise for capturing the blurred edges of clouds. The HRNet model is unable to classify clouds and land features accurately when their spectra and textures are similar, and its limited understanding of context prevents it from accurately identifying thin clouds that are translucent or have blurred boundaries. As for models based on the Transformer architecture, the Transformer structure is primarily designed to process sequential data; consequently, it has poor capabilities for capturing high-frequency local textures, leading to missed and false detections of clouds. Although the model described in this paper does not completely segment the clouds from the original imagery, the method incorporates a module such as CTA, which is specifically designed to perceive multi-scale textural changes within clouds. Through multi-scale convolutions and local spatial statistics-based feature modulation, it is highly sensitive to the unique textural features of clouds, thereby reducing missed detections and improving segmentation accuracy. Furthermore, this paper incorporates an LM module with a global attention mechanism; when identifying a pixel, the model can reference information from pixels at other locations, thereby resulting in fewer missed and false detections compared to other methods. In terms of performance, the segmentation results of this paper are superior, as shown in Figure 10.

The results of the comparative experiments on different models, presented in Table 2, show that our method outperforms other approaches across all five metrics: accuracy, precision, recall, F1 score, and average intersection-over-union. Compared with the baseline model, OCM, the accuracy in this experiment increased by 1.12 percentage points to 94.73 ± 0.18%; precision improved by 2.4 percentage points to 92.28 ± 0.21%; recall rose by 1.24 percentage points to 90.54 ± 0.25%; the F1 score increased by 1.14 percentage points to 89.76 ± 0.22%; and the mloU ratio improved by 1.19 percentage points to 86.65 ± 0.27%. It outperforms OCM by 2.74 percentage points and 1.92 percentage points in terms of BIoU and BF1, respectively. Compared with the traditional DeepLabV3+ [35], UNet, D-LinkNet [36] and HRNet [37] methods, the approach used in this experiment achieves improvements across all metrics. Compared with the three methods—TransUNet [38], Swin-UNet [39] and the Swin-UperNet [40]—used in recent years, the experimental method described in this paper achieved improvements across all metrics; consequently, it outperforms these other methods in all respects and delivers a generally superior segmentation result, as shown in Table 2.

#### 3.3.2. Comparative Experiments of Different Models in Different Scenarios

**Figure 11:**
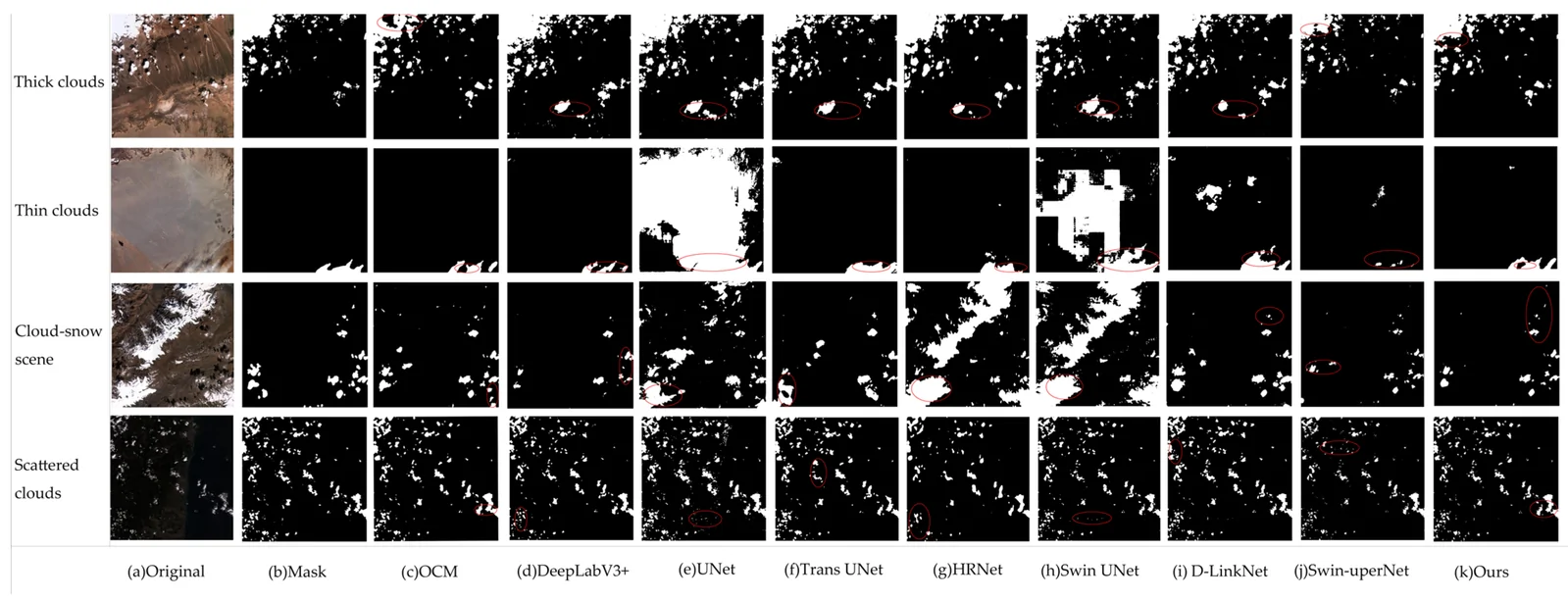
Visual comparison of cloud segmentation results across different scene types: (**a**) Original; (**b**) Mask; (**c**) OCM; (**d**) DeepLabV3+; (**e**) UNet; (**f**) TransUNet; (**g**) HRNet; (**h**) Swin UNet; (**i**) D-LinkNet; (**j**) Swin-uperNet; and (**k**) Ours. The rows show thick clouds, thin clouds, cloud–snow scenes, and scattered clouds.

To provide a more comprehensive demonstration of the segmentation performance of the method described in this paper, Figure 11 presents a comparison of the segmentation results across the various methods. The comparison reveals that, as thick clouds are distributed in clumps across the entire image, high-resolution imagery is prone to variations within the clouds; furthermore, as the original model’s architecture lacks a module specifically designed to detect multi-scale texture changes in such imagery, other comparison models either misclassify bright features as clouds or fail to detect the thick cloud area in the top-left corner. In contrast, following the incorporation of a texture-enhancing attention module with multi-scale convolutional kernels in the experiments described in this paper, the area of missed detection in extensive thick cloud cover was smaller than those of the other comparison models, and the detected clumps of thick cloud were relatively complete. A key characteristic of thin clouds is that, compared to the previous two types of thin clouds, their edges tend to blend more easily with the background. Due to the limited spectral information in the imagery and the model’s limited field of view, the contrast-based model is unable to fully understand the context, resulting in widespread false positives where bright backgrounds are detected as thin clouds. The original OCM model also exhibited some false negatives; in contrast, the model proposed in this paper does not suffer from large-scale false positives, nor does it exhibit severe false negatives. Although the HyTC-Net model still exhibits a small number of false positives and false negatives, its performance stands out compared to other models. In the cloud detection task against a snowy background, where both snow and clouds appear as large areas of white and are easily confused given the limited spectral information, the baseline model OCM and some comparison models can distinguish between most snow and clouds. However, compared to the HyTC-Net network, they still exhibit a relatively high number of false positives, and some comparison models are completely unable to segment clouds and snow. Owing to the global self-attention mechanism of the newly added LM module in this experiment, which possesses contextual reasoning capabilities and can effectively distinguish between clouds and snow, the numbers of false positives and false negatives are significantly reduced. The final category comprises scattered clouds, which are characterized by their highly dispersed and fragmented distribution. Comparative models, lacking multi-scale receptive fields or having a limited number of convolutional kernels, may produce false positives, resulting in numerous instances where the background is incorrectly classified as clouds in the final predictions. In terms of performance, there is little difference between the original OCM model and the model tested in this paper; however, overall, the model tested in this paper outperforms the other comparison models and the baseline model, as shown in Figure 11.

Table 3 shows the model performance across different scenarios. Compared with the OCM model, the HyTC-Net model achieved a 2.5-percentage point increase in accuracy to 95.79 ± 0.16%; a 4.2-percentage point increase in precision to 93.29 ± 0.20%; and a 0.5-percentage point increase in recall to 89.85 ± 0.24%, for the thick cloud scenario. The F1 score increased by 1.2 percentage points to 89.69 ± 0.21%; the mloU rose by 1.4 percentage points to 86.90 ± 0.25%. It obtains a BIoU of 87.26 ± 0.43% and a BF1 of 94.35 ± 0.36%, exceeding the strongest baseline, OCM, by 2.64 and 1.87 percentage points. In the thick cloud scenario, the model used in this experiment demonstrated improvements across all metrics, with the largest increase observed in precision, demonstrating that the model has improved in reducing false negatives; in the thin cloud scenario, the accuracy increased by 0.5 percentage points to 94.04 ± 0.22%; precision by 2.2 percentage points to 91.16 ± 0.26%; and recall by 0.9 percentage points to 90.00 ± 0.29%. The F1 score increased by 0.7 percentage points to 89.20 ± 0.27%; and the mloU rose by 0.93 percentage points to 86.31 ± 0.30%. It achieves 78.54 ± 0.71% BIoU and 88.62 ± 0.59% BF1, corresponding to improvements of 3.08 and 2.25 percentage points over OCM. This indicates that the model described in this paper also outperforms the original model, as well as other traditional and advanced deep learning models, in thin cloud prediction; it effectively avoids misclassification of highly illuminated areas and provides a relatively complete segmentation of the blurred boundaries in thin clouds; in snowy scenes, the accuracy improved by 0.53 percentage points to 93.97 ± 0.28%; the precision improved by 4.39 percentage points to 92.25 ± 0.31%; and the recall improved by 2.1 percentage points to 91.36 ± 0.22%. The F1 score increased by 1.4 percentage points to 90.18 ± 0.30%; and the mloU rose by 2.8 percentage points to 88.44 ± 0.35%. Its BIoU and BF1 reach 83.18 ± 0.58% and 91.76 ± 0.47%, surpassing OCM by 2.61 and 1.92 percentage points. Against the high-brightness background of snowy terrain, the HyTC-Net model’s metrics were better than those predicted by the original model, demonstrating that the model used in this experiment is better able to distinguish clouds against a high-brightness background. Under the ‘scattered clouds’ category, accuracy improved by 1.3 percentage points to 95.19 ± 0.20%; precision by 2.16 percentage points to 92.42 ± 0.25%; recall by 1.5 percentage points to 90.95 ± 0.27%; the F1 score by 2 percentage points to 90.61 ± 0.24%; and the mloU by 0.6 percentage points to 85.95 ± 0.28%. It obtains 80.50 ± 0.76% BIoU and 89.29 ± 0.63% BF1, with gains of 2.63 and 1.34 percentage points. Consequently, the method proposed in this paper demonstrates improvements across all metrics compared to the original model and other benchmark models.

As reported in Table 3, HyTC-Net achieves higher BIoU and BF1 scores than OCM across the evaluated scene categories, indicating improved boundary delineation on these test subsets, achieving a 0.9–10.7-percentage point improvement in accuracy and a 0.35–19.4-percentage point improvement in precision compared with other models. For thin cloud types, it also avoids a large number of false positives caused by highly reflective ground features, as well as the issue of blurred cloud edge predictions, achieving a 0.4–9.5-percentage point increase in accuracy and a 0.5–8.3-percentage point increase in precision compared to other models. In snowy scenes, the HyTC-Net model reduces false-positive predictions in the illustrated examples, thereby avoiding false positives; its accuracy is 0.21–8.46 percentage points higher than those of other models, while its precision is 0.31–10.28 percentage points higher. With regard to patchy clouds, this study achieves a relatively comprehensive detection of such features in the imagery, with an accuracy rate 0.23–9.78 percentage points higher and a precision 0.72–8.92 percentage points higher than other models.

Overall, the HyTC-Net model demonstrated improved performance across all four cloud type segmentations, showing modest improvements compared to some of the benchmark models. When considering the five metrics collectively, the model outperformed the benchmark models, and the segmentation results from this study were also superior to those of other comparative experiments. Consequently, the model tested in this study achieved improved segmentation performance across all cloud categories, as shown in Table 3.

### 3.4. Ablation Experiment

#### 3.4.1. Module Ablation Experiment

To evaluate the individual and combined contributions of CTA, L-WASPP, LM, and DS to cloud segmentation performance, we conducted ablation experiments with different module configurations. When L-WASPP, LM and DS were added individually, performance remained stable or showed slight improvement; when introduced individually, the CTA module improves BIoU and BF1, while recall decreases slightly compared with the OCM baseline. These results suggest that CTA contributes to boundary delineation, although its standalone use does not improve cloud-pixel sensitivity. In the ablation experiments, while some combinations did yield improvements of 0.03–0.75 percentage points in precision and 0.09–3.56 percentage points in recall, with F1, mIoU remained almost on par with the baseline model, and adding CTA to this configuration further increased BIoU by 1.25 percentage points and BF1 by 0.65 percentage points. These improvements were not as significant as the enhancement in segmentation performance achieved when all four modules were combined. The CTA module primarily improves the model’s segmentation performance for highly illuminated objects and clouds, effectively resolving the issue of confusion between these features; the L-WASPP module utilizes convolutions with varying expansion ratios to capture contextual information in parallel; the LM module employs a self-attention mechanism to compute relationships between any two positions, establishing long-range dependencies that enable the model to better understand the overall structure; and the DS module ensures that gradients are effectively propagated through each network layer, thereby mitigating the vanishing gradient problem. After combining these four modules in pairs and in groups of three, the model’s ability to recognize the edges of thin and patchy clouds, as well as its segmentation capabilities for highly illuminated ground objects and clouds, was enhanced.

Compared to the baseline model, accuracy increased by 1.12 percentage points to 94.73 ± 0.18%; precision by 2.4 percentage points to 92.28 ± 0.21%; recall by 1.24 percentage points to 90.54 ± 0.25%; the F1 increased by 1.14 percentage points to 89.76 ± 0.22%, and the mloU rose by 1.19 percentage points to 86.65 ± 0.27%, with a BIoU of 82.37 ± 0.52% and a BF1 of 91.08 ± 0.44%, exceeding the OCM baseline by 2.74 and 1.92 percentage points, respectively. Specifically, configurations 1–4 evaluate the individual modules, configurations 5–10 evaluate two-module combinations, and configurations 11–14 evaluate three-module combinations. These results reveal metric-dependent effects, rather than uniform improvements across all configurations. Table 4 presents the data from the module ablation experiments.

#### 3.4.2. Loss Function Ablation Experiments

In the field of semantic segmentation, particularly in highly challenging applications such as cloud detection, the selection and design of the loss function are key factors in determining the model’s optimization path and ultimate segmentation performance. The complexity of this decision stems from the inherent physical characteristics of cloud objects and their manifestation in remote sensing imagery. Specifically, clouds typically exhibit high morphological variability, amorphous structures, and blurred, indistinct boundaries. Consequently, cloud detection tasks are generally plagued by severe class imbalance between foreground and background categories, meaning that cloud pixels may constitute only a very small proportion of the entire image. Against this backdrop, traditional loss functions—such as the standard CE, Dice, loU and Boun—often struggle to achieve global optimization. However, newly improved composite loss functions effectively address this issue; this ablation study demonstrates the results of these four loss functions when used individually or in combination.

As shown in Table 5, the proposed loss configuration achieves the highest mean scores across all seven evaluation metrics. Compared with the original OCM configuration, accuracy increases by 1.12 percentage points to 94.73 ± 0.18%; precision by 2.40 percentage points to 92.28 ± 0.21%; recall by 1.24 percentage points to 90.54 ± 0.25%; F1 score by 1.14 percentage points to 89.76 ± 0.22%; and mIoU by 1.19 percentage points to 86.65 ± 0.27%. BIoU and BF1 reach 82.37 ± 0.52% and 91.08 ± 0.44%, exceeding the OCM baseline by 2.74 and 1.92 percentage points, respectively. These improvements indicate better region segmentation and boundary delineation. Although some intermediate loss configurations exhibit lower precision or recall than the baseline, their BIoU and BF1 scores consistently increase. The final configuration achieves the highest mean precision and recall, while also providing the best boundary metrics among the evaluated configurations. The DT loss function adaptively adjusts hyperparameters to address false positives and false negatives at different stages of training, whereas the baseline model’s Dice and IoU loss functions treat FP and FN equally. The TAB loss function utilizes texture information to accurately segment complex texture boundaries; compared to the original Boun function, it is less susceptible to interference from internal textures and blurred regions within the image. The newly added Con places greater emphasis on intrinsic stability than other loss functions, thereby enhancing the stability of the model’s segmentation performance relative to the OCM loss function.

The data in Table 5 pertain to ablation experiments on the loss functions. Changes 1, 2, and 3 demonstrate the effectiveness of the newly introduced DT and TAB loss functions, as well as the Con, on the model. Changes 4 and 5 involve combining the loss functions, ultimately concluding that the fusion of the four loss functions yields the best results. The Change 1 to Change 5 experiments shown in Table 5 were conducted using the same HyTC-Net architecture, data split, and training settings; only the loss function configuration was varied.

## 4. Discussion

This paper presents a cloud detection experiment conducted on imagery acquired by the ZY-3 satellite. The HyTC-Net deep learning network, which utilizes the ConvNext-Tiny backbone, was designed to address the issues encountered by the original OCM model—namely, false positives and false negatives regarding cloud boundaries; thin clouds; patchy clouds; and imagery with snow-covered backgrounds—in the high-resolution images from ZY-3 missions 01 and 02. Cloud detection and segmentation technology has evolved from physical rules and traditional machine learning to deep learning. Traditional methods are limited by rigid rules and reliance on manually extracted features; early deep learning approaches, while capable of automatically learning features, were costly and had limited understanding of complex scenes; although current deep learning models are now mature, there is still room for improvement in their segmentation performance when handling texture and edge details in high-resolution imagery, as well as in complex scenes with limited spectral information. Consequently, to maintain constant experimental conditions, this experiment retained the eight data augmentation techniques from the original OCM model during the data pre-processing stage. By incorporating multiple scene classifications into the dataset, it has been demonstrated that the model exhibits good segmentation performance across different cloud types in ZY-3 satellite imagery. The addition of a custom CTA module, which combines channel, texture and frequency attention mechanisms, enables the capture of cloud texture information at multiple scales, while also integrating three modules: L-WASPP, LM and DS. Optimizations were carried out at three levels—multi-scale feature extraction, global context modeling and DS training—significantly improving the completeness and accuracy of cloud segmentation. Furthermore, TAB loss is introduced as a modification of the original Boun loss, and DT loss is formulated through the modification and combination of Dice and IoU loss terms. Together, these components form a training objective designed to improve region overlap and cloud boundary delineation. The newly added Con improves the model’s stability in segmentation performance, while the integration of the CE cross-entropy loss function assigns appropriate weights to each loss function to monitor model training and drive the neural network to learn. By utilizing the AdamW optimizer via backpropagation to update parameters and minimize error, the HyTC-Net model can segment thick clouds, thin clouds with blurred boundaries, clouds against snowy backgrounds, and patchy clouds with considerable accuracy.

Overall, the HyTC-Net model achieved a 1.12-percentage point improvement in accuracy compared to the original OCM model, reaching 94.73 ± 0.18%; a 2.4-percentage point increase in precision to 92.28 ± 0.21%; a 1.24-percentage point rise in recall to 90.54 ± 0.25%; a 1.14-percentage point increase in F1 score to 89.76 ± 0.22%; and a 1.19-percentage point rise in the mloU to 86.65 ± 0.27%. It outperforms OCM by 2.74 percentage points to 82.37 ± 0.52%, and 1.92 percentage points to 91.08 ± 0.44%, in terms of BIoU and BF1, respectively. In cases where high-resolution imagery results in uneven texture within thick clouds, the HyTC-Net model reduces false negatives compared to previous models; it achieves more precise segmentation at the blurred edges of thin clouds and ground features; under snowy backgrounds, it minimizes the misclassification of bright snow areas as clouds compared to existing models; and, for fragmented cloud types, the HyTC-Net model generally produces more complete predictions. The model designed in this experiment demonstrates a robust foundation for feature extraction. However, due to the large size of the input images and the fact that the images underwent eight rounds of data augmentation, the model’s training took a significant amount of time.

Although the accuracy of this model, developed for ZY-3 satellite imagery, has been improved to a certain extent, improving the generalization ability of HyTC-Net is an important direction for future research. Differences in sensor characteristics, spatial resolution, acquisition conditions, and geographical environments may alter the appearances of clouds and background surfaces, potentially affecting the transferability of the model. Future work will therefore evaluate HyTC-Net across independent datasets using cross-sensor, cross-region, and cross-season testing protocols. Further work is required to enhance its robustness before it can be deployed on a large scale in engineering production, ensuring it delivers superior performance in cloud detection tasks across a wider range of satellite imagery. Particular attention will be given to thin clouds and cloud boundaries over snow, ice, bright buildings, and other highly reflective surfaces, where cloud–background ambiguity remains challenging. Expanding the training data to include a wider range of cloud textures and surface conditions, together with physically plausible spectral and spatial augmentation, may improve robustness to these variations.

## 5. Conclusions

This paper primarily addresses the following issues with the original OCM model: incomplete recognition of thin clouds with blurred boundaries in images from the ZY-3 satellite; discontinuous segmentation of thick and fragmented clouds; confusion between cloud and snow recognition against high-brightness backgrounds, such as snow-covered ground; and the vanishing gradient problem as training progresses. In the HyTC-Net model, the self-attention mechanism of the Transformer in the LM module enables more complete segmentation of clouds with varying shapes; the CTA module uses multi-scale convolutions and local statistical texture analysis to detect energy distribution, thereby reducing confusion between clouds and snow, as well as the issue of incomplete thin cloud segmentation; and the multi-scale receptive fields of the L-WASPP module result in more complete cloud segmentation and clearer cloud boundaries. In this experiment, the training results of the HyTC-Net model showed a 1.12% increase in accuracy and a 2.4% increase in precision compared to the original model. Unlike the baseline model, the HyTC-Net model, under conditions of limited spectral information and high-resolution imagery, preserves the edge details of thin clouds, clearly identifies cloud features even against the high-brightness background of snow-covered terrain, and continuously segments both thick and patchy clouds. For satellite imagery of the ZY-3 type, by integrating modules that are sensitive to texture and highly responsive to contextual features, the model establishes a global understanding of context, enabling it to learn the detailed characteristics of different types of cloud textures. The proposed CTA module combines channel attention, multi-scale texture attention, and modulation based on local spatial statistics to enrich cloud-related feature representations. The F-branch preserves the spatial alignment of local variation descriptors and complements the other branches, whilst the final cloud classification results are generated by the segmentation network. This enables the model to identify pixel locations and cloud presence with greater precision. The addition of these modules enables the model to learn the detailed characteristics of different types of cloud textures in ZY-3 satellite imagery. This demonstrates superior performance compared to the baseline model in cloud detection for ZY-3 satellite imagery.

Future work will focus on improving the generalization of HyTC-Net across sensors, geographical regions, seasons, and spatial resolutions. This will involve expanding the diversity of the training data and investigating self-supervised pretraining, domain adaptation, and boundary-aware learning. An independent multimodal assessment will be carried out, focusing in particular on thin cloud layers and clouds situated against highly reflective backgrounds, in order to evaluate the model’s robustness and transferability under a wider range of remote sensing conditions.

## Figures and Tables

**Table 1:** Experimental environment.

Item	Parameters
Operating System	Windows10
Graphics card model	NVIDIA Quadro P6000
Graphics card memory	24 GB
Processor	Intel(R) Xeon(R) Silver 4116 CPU @ 2.10 GHz
Deep learning frameworks	Pytorch2.5.1 + cuda11.8

**Table 2:** Experimental data comparing different models.

Method	Accuracy	Precision	Recall	F1	mIoU	BIoU	BF1	Parameter
OCM	93.61 ± 0.28	89.88 ± 0.32	89.30 ± 0.35	88.62 ± 0.33	85.46 ± 0.30	79.63 ± 0.58	89.16 ± 0.46	7,840,000
DeepLabV3+	89.11 ± 0.58	90.79 ± 0.45	89.11 ± 0.62	88.57 ± 0.55	82.04 ± 0.68	69.42 ± 0.86	81.73 ± 0.71	**5,220,000**
UNet	90.46 ± 0.75	75.76 ± 0.88	77.25 ± 0.95	72.78 ± 1.10	73.90 ± 0.92	71.16 ± 0.79	82.94 ± 0.65	31,030,000
TransUNet	94.57 ± 0.45	72.77 ± 0.93	80.63 ± 0.78	75.32 ± 0.85	77.35 ± 0.80	74.81 ± 0.68	85.47 0.57	105,270,000
HRNet	88.12 ± 0.65	78.25 ± 0.72	74.61 ± 0.81	76.33 ± 0.77	73.52 ± 0.85	73.58 ± 0.73	84.62 ± 0.61	9,640,000
SwinUNet	84.88 ± 0.82	74.32 ± 1.05	84.50 ± 0.90	73.71 ± 1.21	69.24 ± 1.35	75.69 ± 0.72	86.08 ± 0.60	27,170,000
D-LinkNet	93.25 ± 0.48	81.20 ± 0.65	83.14 ± 0.59	80.56 ± 0.61	79.43 ± 0.75	76.94 ± 0.61	87.02 ± 0.51	31,440,000
Swin-UperNet	94.09 ± 0.38	91.82 ± 0.49	84.53 ± 0.55	88.02 ± 0.47	78.61 ± 0.65	78.12 ± 0.64	88.05 ± 0.53	6,130,000
Ours	**94.73 ± 0.18**	**92.28 ± 0.21**	**90.54 ± 0.25**	**89.76 ± 0.22**	**86.65 ± 0.27**	**82.37 ± 0.52**	**91.08 ± 0.44**	59,090,000

Note: The bolded values represent the best results in each column. Higher values indicate better performance across all evaluation metrics, and fewer parameters are generally better.

**Table 3:** Experimental data for different cloud types across different models.

	Indicator	Thick Clouds	Thin Clouds	Clouds–SnowScene	Scattered Clouds
OCM	Accuracy	93.25 ± 0.31	93.56 ± 0.35	93.44 ± 0.42	93.86 ± 0.33
	Precision	89.03 ± 0.40	88.97 ± 0.45	87.86 ± 0.51	90.26 ± 0.41
	Recall	89.37 ± 0.38	89.11 ± 0.41	89.26 ± 0.48	89.46 ± 0.39
	F1	88.49 ± 0.39	88.59 ± 0.43	88.74 ± 0.49	88.66 ± 0.40
	mIoU	85.52 ± 0.42	85.38 ± 0.48	85.62 ± 0.53	85.32 ± 0.45
	BIoU	84.62 ± 0.51	75.46 ± 0.82	80.57 ± 0.66	77.87 ± 0.88
	BF1	92.48 ± 0.42	86.37 ± 0.68	89.84 ± 0.55	87.95 ± 0.73
DeepLabV3+	Accuracy	89.23 ± 0.45	89.13 ± 0.51	88.90 ± 0.60	89.18 ± 0.48
	Precision	90.67 ± 0.38	90.54 ± 0.42	91.89 ± 0.45	90.06 ± 0.41
	Recall	89.08 ± 0.65	89.24 ± 0.72	89.47 ± 0.80	88.65 ± 0.62
	F1	88.78 ± 0.51	88.36 ± 0.58	88.43 ± 0.63	88.71 ± 0.55
	mIoU	82.13 ± 0.55	81.96 ± 0.62	81.89 ± 0.68	82.07 ± 0.58
	BIoU	75.83 ± 0.78	63.58 ± 1.16	70.15 ± 0.98	66.42 ± 1.24
	BF1	86.25 ± 0.66	76.42 ± 0.97	82.36 ± 0.82	78.63 ± 1.02
UNet	Accuracy	90.61 ± 0.65	90.25 ± 0.75	90.42 ± 0.71	90.56 ± 0.68
	Precision	75.32 ± 0.88	74.96 ± 0.95	75.87 ± 0.92	76.89 ± 0.85
	Recall	77.67 ± 0.75	77.31 ± 0.82	76.25 ± 0.88	77.58 ± 0.78
	F1	73.09 ± 0.81	72.26 ± 0.88	72.48 ± 0.79	73.29 ± 0.66
	mIoU	74.27 ± 0.53	73.65 ± 0.69	73.57 ± 0.57	74.11 ± 0.48
	BIoU	77.36 ± 0.72	65.72 ± 1.08	71.89 ± 0.91	68.15 ± 1.13
	BF1	87.41 ± 0.59	77.83 ± 0.89	83.58 ± 0.72	79.74 ± 0.94
TransUNet	Accuracy	94.89 ± 0.25	93.64 ± 0.38	93.41 ± 0.45	94.96 ± 0.29
	Precision	72.69 ± 1.12	72.88 ± 0.86	71.97 ± 1.35	73.54 ± 1.15
	Recall	80.98 ± 0.55	79.62 ± 0.68	79.45 ± 0.75	81.29 ± 0.60
	F1	75.45 ± 0.80	74.94 ± 0.92	75.06 ± 1.01	75.83 ± 0.85
	mIoU	77.20 ± 0.85	76.88 ± 0.99	77.46 ± 0.95	77.86 ± 0.88
	BIoU	80.49 ± 0.61	69.83 ± 0.94	75.62 ± 0.79	72.26 ± 0.99
	BF1	89.56 ± 0.51	81.26 ± 0.78	86.14 ± 0.66	84.08 ± 0.82
HRNet	Accuracy	88.56 ± 0.53	87.43 ± 0.65	87.81 ± 0.68	88.68 ± 0.55
	Precision	79.14 ± 0.71	78.00 ± 0.82	77.30 ± 0.88	78.56 ± 0.75
	Recall	74.82 ± 0.68	73.16 ± 0.78	74.56 ± 0.85	74.10 ± 0.71
	F1	76.92 ± 0.69	76.14 ± 0.80	75.31 ± 0.86	76.47 ± 0.73
	mIoU	73.82 ± 0.78	72.96 ± 0.90	73.43 ± 0.95	73.90 ± 0.81
	BIoU	79.25 ± 0.65	68.41 ± 1.01	74.36 ± 0.85	71.09 ± 1.06
	BF1	88.73 ± 0.54	80.35 ± 0.84	85.27 ± 0.70	83.16 ± 0.88
SwinUNet	Accuracy	85.08 ± 0.73	84.54 ± 0.85	84.49 ± 0.92	85.41 ± 0.80
	Precision	74.00 ± 0.95	73.97 ± 1.02	74.66 ± 0.96	74.76 ± 0.98
	Recall	84.89 ± 0.85	84.26 ± 0.95	84.33 ± 1.05	84.52 ± 0.74
	F1	74.84 ± 0.90	73.60 ± 0.98	73.41 ± 1.07	72.99 ± 0.93
	mIoU	71.27 ± 0.78	68.66 ± 0.67	67.58 ± 0.95	69.45 ± 0.81
	BIoU	81.16 ± 0.64	70.92 ± 0.98	76.48 ± 0.83	73.18 ± 1.02
	BF1	90.12 ± 0.52	82.14 ± 0.81	86.72 ± 0.68	85.34 ± 0.84
D-LinkNet	Accuracy	94.79 ± 0.30	91.94 ± 0.48	92.34 ± 0.55	93.93 ± 0.41
	Precision	84.59 ± 0.55	80.77 ± 0.68	76.36 ± 0.75	81.08 ± 0.65
	Recall	84.44 ± 0.45	81.69 ± 0.58	82.15 ± 0.65	84.28 ± 0.51
	F1	82.63 ± 0.50	78.91 ± 0.63	80.54 ± 0.70	80.16 ± 0.58
	mIoU	79.17 ± 0.65	77.34 ± 0.75	78.89 ± 0.80	82.32 ± 0.58
	BIoU	82.37 ± 0.55	72.16 ± 0.87	77.83 ± 0.71	74.92 ± 0.91
	BF1	90.86 ± 0.45	83.48 ± 0.72	87.65 ± 0.59	86.09 ± 0.76
Swin-UperNet	Accuracy	94.78 ± 0.28	93.39 ± 0.36	93.76 ± 0.40	94.43 ± 0.32
	Precision	92.94 ± 0.35	90.61 ± 0.41	91.03 ± 0.46	91.70 ± 0.38
	Recall	85.15 ± 0.48	82.45 ± 0.55	84.11 ± 0.60	84.41 ± 0.51
	F1	88.87 ± 0.41	86.93 ± 0.48	88.96 ± 0.53	86.85 ± 0.44
	mIoU	77.85 ± 0.60	76.92 ± 0.73	78.13 ± 0.68	81.45 ± 0.55
	BIoU	83.45 ± 0.57	73.85 ± 0.90	78.94 ± 0.74	76.24 ± 0.95
	BF1	91.72 ± 0.47	84.76 ± 0.74	88.43 ± 0.61	87.28 ± 0.79
Ours	Accuracy	95.79 ± 0.16	94.04 ± 0.22	93.97 ± 0.28	95.19 ± 0.20
	Precision	93.29 ± 0.20	91.16 ± 0.26	92.25 ± 0.31	92.42 ± 0.25
	Recall	89.85 ± 0.24	90.00 ± 0.29	91.36 ± 0.22	90.95 ± 0.27
	F1	89.69 ± 0.21	89.20 ± 0.27	90.18 ± 0.30	90.61 ± 0.24
	mIoU	86.90 ± 0.25	86.31 ± 0.30	88.44 ± 0.35	85.95 ± 0.28
	BIoU	87.26 ± 0.43	78.54 ± 0.71	83.18 ± 0.58	80.50 ± 0.76
	BF1	94.35 ± 0.36	88.62 ± 0.59	91.76 ± 0.47	89.29 ± 0.63

**Table 4:** Results of module ablation experiments. √ indicates that the corresponding module is included, whereas × indi-cates that it is excluded. Bold values indicate the highest mean values in their respective metric columns.

Change	CTA	L-WASPP	LM	DS	Accuracy	Precision	Recall	F1	mIoU	BIoU	BF1
OCM	×	×	×	×	93.61 ± 0.28	89.88 ± 0.32	89.30 ± 0.35	88.62 ± 0.33	85.46 ± 0.30	79.63 ± 0.58	89.16 ± 0.46
1	√	×	×	×	93.92 ± 0.18	91.59 ± 0.28	88.28 ± 0.34	88.02 ± 0.25	85.47 ± 0.21	79.71 ± 0.55	89.24 ± 0.44
2	×	√	×	×	93.65 ± 0.20	89.97 ± 0.31	89.68 ± 0.29	87.44 ± 0.26	84.77 ± 0.24	79.48 ± 0.57	89.08 ± 0.45
3	×	×	√	×	93.93 ± 0.19	89.66 ± 0.33	88.26 ± 0.30	88.20 ± 0.24	85.52 ± 0.22	79.86 ± 0.54	89.39 ± 0.43
4	×	×	×	√	93.71 ± 0.21	87.96 ± 0.36	87.51 ± 0.35	87.74 ± 0.28	85.02 ± 0.26	79.36 ± 0.59	88.97 ± 0.47
5	√	√	×	×	93.99 ± 0.17	89.92 ± 0.29	86.29 ± 0.32	88.08 ± 0.23	85.49 ± 0.20	80.02 ± 0.53	89.55 ± 0.42
6	√	×	√	×	94.05 ± 0.16	93.34 ± 0.25	88.69 ± 0.28	86.55 ± 0.22	85.81 ± 0.19	80.41 ± 0.51	89.86 ± 0.41
7	√	×	×	√	93.84 ± 0.21	91.93 ± 0.27	83.09 ± 0.38	87.28 ± 0.25	85.02 ± 0.23	79.94 ± 0.56	89.43 ± 0.45
8	×	√	√	×	93.74 ± 0.20	89.74 ± 0.30	85.31 ± 0.33	87.56 ± 0.24	84.92 ± 0.27	79.68 ± 0.57	89.20 ± 0.46
9	×	√	×	√	93.50 ± 0.23	91.08 ± 0.25	85.15 ± 0.35	88.20 ± 0.27	84.48 ± 0.28	79.83 ± 0.55	89.37 ± 0.44
10	×	×	√	√	93.64 ± 0.21	92.39 ± 0.26	87.73 ± 0.31	87.67 ± 0.24	84.91 ± 0.25	80.18 ± 0.52	89.71 ± 0.42
11	√	√	√	×	94.15 ± 0.15	90.54 ± 0.24	85.14 ± 0.29	88.47 ± 0.21	85.34 ± 0.18	80.56 ± 0.49	90.02 ± 0.40
12	√	√	×	√	93.94 ± 0.18	89.23 ± 0.28	86.96 ± 0.30	88.19 ± 0.23	85.52 ± 0.20	80.21 ± 0.53	89.69 ± 0.43
13	√	×	√	√	93.76 ± 0.19	87.29 ± 0.34	88.46 ± 0.23	87.97 ± 0.25	85.22 ± 0.22	80.35 ± 0.50	89.88 ± 0.41
14	×	√	√	√	94.36 ± 0.14	90.99 ± 0.23	89.51 ± 0.25	89.38 ± 0.19	85.75 ± 0.17	81.12 ± 0.47	90.43 ± 0.39
Ours	√	√	√	√	**94.73 ± 0.18**	**92.28 ± 0.21**	**90.54 ± 0.25**	**89.76 ± 0.22**	**86.65 ± 0.27**	**82.37 ± 0.52**	**91.08 ± 0.44**

**Table 5:** Performance comparison of different loss function combinations. √ indicates that the corresponding loss term is included, whereas × indicates that it is excluded. Bold values indicate the highest mean values in their respective metric columns.

Change	CE	Dice	IoU	Boun	DT	TAB	Con	Accuracy	Precision	Recall	F1	mIoU	BIoU	BF1
OCM	√	√	√	√	×	×	×	93.61 ± 0.28	89.88 ± 0.32	89.30 ± 0.35	88.62 ± 0.33	85.46 ± 0.30	79.63 ± 0.58	89.16 ± 0.46
1	√	×	×	√	√	×	×	93.97 ± 0.19	90.74 ± 0.29	89.81 ± 0.28	88.53 ± 0.25	84.10 ± 0.23	79.74 ± 0.56	89.29 ± 0.44
2	√	√	√	×	×	√	×	93.63 ± 0.21	89.82 ± 0.32	89.76 ± 0.32	88.39 ± 0.27	85.91 ± 0.24	80.06 ± 0.54	89.52 ± 0.43
3	√	√	√	√	×	×	√	93.75 ± 0.20	89.47 ± 0.34	88.35 ± 0.33	88.94 ± 0.26	85.62 ± 0.23	80.15 ± 0.53	89.64 ± 0.42
4	√	×	×	×	√	×	×	89.90 ± 0.28	89.90 ± 0.28	88.84 ± 0.29	88.60 ± 0.23	85.06 ± 0.20	80.24 ± 0.52	89.71 ± 0.41
5	√	×	×	×	√	√	×	91.76 ± 0.24	89.67 ± 0.26	89.67 ± 0.26	89.08 ± 0.20	86.15 ± 0.18	81.05 ± 0.49	90.18 ± 0.39
Ours	√	×	×	×	√	√	√	**94.73 ± 0.18**	**92.28 ± 0.21**	**90.54 ± 0.25**	**89.76 ± 0.22**	**86.65 ± 0.27**	**82.37 ± 0.52**	**91.08 ± 0.44**

## Data Availability

The ZY-3 data used in this study are subject to institutional restrictions; processed subsets are available from the corresponding author upon reasonable request, with permission from the data owner.

## Abbreviations

The following abbreviations are used in this manuscript:

CTA	CloudTextureAttention
LM	LightweightMobileViT
OCM	OmniCloudMask
HyTC-Net	HybridTexture-aware Cloud Detection Network
NDVI	Normalized Difference Vegetation Index
L-WASPP	Lightweight ASPP
DS	Deep Supervision
DT	Dynamic Tversky
TAB	Texture-Aware Boundary
FNN	Feedforward neural network
MLP	multilayer perceptron
mIoU	Mean Intersection-over-Union
Boun	Boundary loss
BIoU	Boundary IoU
BF1	Boundary F1
